# Chronic inflammation induces telomere dysfunction and accelerates ageing in mice

**DOI:** 10.1038/ncomms5172

**Published:** 2014-06-24

**Authors:** Diana Jurk, Caroline Wilson, João F. Passos, Fiona Oakley, Clara Correia-Melo, Laura Greaves, Gabriele Saretzki, Chris Fox, Conor Lawless, Rhys Anderson, Graeme Hewitt, Sylvia LF Pender, Nicola Fullard, Glyn Nelson, Jelena Mann, Bart van de Sluis, Derek A. Mann, Thomas von Zglinicki

**Affiliations:** 1 Institute for Ageing and Health, Newcastle University, NE4 5PL, UK; 2 Fibrosis Laboratory, Liver Group, Institute of Cellular Medicine, Newcastle University, Newcastle upon Tyne NE2 4HH, UK; 3 Mitochondrial Research Group, Institute for Ageing and Health, Newcastle University, Newcastle upon Tyne NE2 4HH, UK; 4 Institute for Cell and Molecular Biosciences, Newcastle University, Catherine Cookson Building, Framlington Place, Newcastle Upon Tyne NE2 4HH, UK; 5 Faculty of Medicine, University of Southampton. Mailpoint 813, Sir Henry Wellcome Laboratories, Southampton General Hospital, Tremona Road, Southampton SO16 6YD, UK; 6 Molecular Genetics Laboratory, Department of Pathology and Medical Biology, University of Groningen, University Medical Center Groningen, 9713 AV Groningen, The Netherlands; 7 These authors contributed equally to this work

## Abstract

Chronic inflammation is associated with normal and pathological ageing. Here we show that chronic, progressive low-grade inflammation induced by knockout of the *nfkb1* subunit of the transcription factor NF-κB induces premature ageing in mice. We also show that these mice have reduced regeneration in liver and gut. *nfkb1*^*−/−*^ fibroblasts exhibit aggravated cell senescence because of an enhanced autocrine and paracrine feedback through NF-κB, COX-2 and ROS, which stabilizes DNA damage. Preferential accumulation of telomere-dysfunctional senescent cells in *nfkb1*^*−/−*^ tissues is blocked by anti-inflammatory or antioxidant treatment of mice, and this rescues tissue regenerative potential. Frequencies of senescent cells in liver and intestinal crypts quantitatively predict mean and maximum lifespan in both short- and long-lived mice cohorts. These data indicate that systemic chronic inflammation can accelerate ageing via ROS-mediated exacerbation of telomere dysfunction and cell senescence in the absence of any other genetic or environmental factor.

## Abstract (web summary)

Many age-related diseases are associated with chronic inflammation. Here Jurk *et al*. use a mouse model of chronic, low-grade inflammation to support a model by which such inflammation promotes a vicious cycle of oxidative stress, telomere dysfunction and cell senescence that accelerates the ageing process.

## Graphical abstract

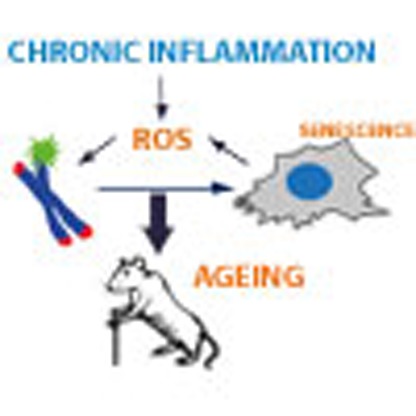

Many age-related diseases and ageing itself are closely associated with low-level chronic inflammation^1^,^2^. The essential question whether chronic inflammation is causal for the development of age-related disease and in normal ageing has only very recently begun to be experimentally addressed. Recent data show that inflammation may influence systemic ageing via neuro-endocrine signalling^3^. On a tissue level, inflammation might contribute to ageing in mouse skin^4^ and in DNA damage-driven progeria mouse models^5^,^6^,^7^, suggesting synergistic interactions between DNA damage responses (DDRs) and inflammatory signals. However, the molecular and cellular mechanisms of such interactions during ageing are still not understood. Moreover, progerias are accompanied by severe pathologies and the question how far such ageing models resemble normal ageing remains unresolved^8^.

Telomere dysfunction inducing a persistent DDR is a major cause of cellular senescence^9^. Severe telomere dysfunction is induced by telomere shortening in late-generation telomerase (*terc*^*−/−*^) knockout mice, where it compromises the function of tissue-specific stem and progenitor cells, limits tissue regenerative capacity and accelerates ageing^10^. However, telomere shortening is just one mechanism to ‘uncap’ telomeres. Senescent cells harbouring dysfunctional telomeres, which are recognized by persistent telomere-associated DNA damage foci (TAF), accumulate even in tissues of ageing mice with long telomeres, suggesting that telomere dysfunction may contribute to age-related decline in tissue function and regeneration during normal ageing of mice^11^. Senescent cells activate hyper-production of reactive oxygen species (ROS)^12^ and secrete bioactive, frequently pro-inflammatory peptides (the so-called senescence-associated secretory phenotype (SASP) or senescent-messaging secretome)^13^,^14^,^15^,^16^. In senescent fibroblasts and in oncogene-induced senescence, the SASP is closely controlled by signalling through NF-κB^16^,^17^. Both senescence-associated ROS^12^ and NF-κB-driven pro-inflammatory cytokines, especially IL-6 and IL-8 (refs 13,14), contribute to positive feedback loops that stabilize oncogene- or stress-induced senescence. In a specific progeria mouse model, targeted ablation of senescent cells has been sufficient to delay age-associated degenerative loss of function in multiple tissues^18^. However, it is still unknown how cell senescence might contribute to organism ageing.

We hypothesize that chronic low-grade inflammation might enhance telomere dysfunction by increasing ROS-mediated DNA damage and thus accelerate accumulation of senescent cells, initiating a ‘circulus vitiosus’ in which cell senescence aggravates chronic inflammation, limits tissue regeneration and accelerates ageing.

To test this hypothesis, we utilized a mouse model of chronic low-level inflammation, the *nfkb1*^*−/−*^ mouse that lacks expression of the p105 and p50 NF-κB proteins. NF-κB is the cardinal transcriptional regulator of inflammation-related genes including pro-inflammatory interleukins, chemokines, cytokines, adhesion molecules and others, and is itself activated by pro-inflammatory, stress and cell senescence signals^19^. NF-κB controls inflammatory gene expression through the activities of its five-subunit components (RelA, RelB, c-Rel, p105/p50 and p100/p52), which operate as homo- or heterodimers. The classic pro-inflammatory NF-κB is the RelA:p50 heterodimer with RelA being required for stimulation of target gene transcription. By contrast the homodimer (p50:p50) is an active repressor of pro-inflammatory gene transcription^20^. This repressive function is at least in part attributed to the absence of a transactivation domain in p50 and the ability of p50:p50 to recruit histone deactylase 1 (HDAC1) to a subset of κB motifs^20^,^21^. The p50:p50:HDAC1 competes with RelA-containing dimers for κB motifs and actively represses transcription by deacetylation of histones. As a consequence *nfkb1*^*−/−*^ mice, which are unable to form p50:p50 but can still generate RelA-containing NF-κB dimers, show enhanced responses to inflammatory stimuli^21^,^22^ and are thought to have a low-level elevated inflammatory phenotype^20^.

Our results show that chronic inflammation aggravates telomere dysfunction and cell senescence, decreases regenerative potential in multiple tissues and accelerates ageing of mice. Anti-inflammatory or antioxidant treatment, specifically COX-2 inhibition, rescued telomere dysfunction, cell senescence and tissue regenerative potential, indicating that chronic inflammation may accelerate ageing at least partially in a cell-autonomous manner via COX-2-dependent hyper-production of ROS.

## Results

### Progressive chronic inflammation in *nfkb1*^*−/−*^ mice

**Figure 1:**
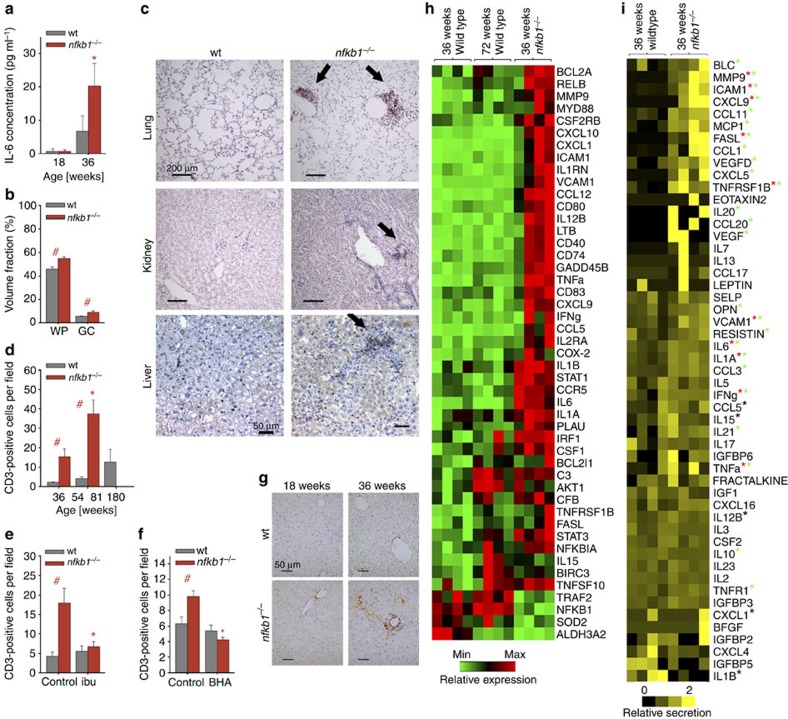
Loss of *nfkb1* induces an inflammatory phenotype in mice. All data are mean (M) ±s.e.m., 5–6 animals per group, if not otherwise indicated. Significant differences (ANOVA, *P*<0.05) to young animals are indicated by *, and between wt and *nfkb1*^*−/−*^ strains at the same age group by #. (**a**) IL-6 levels in blood plasma as measured by ELISA at the indicated ages. (**b**) White pulp (WP) and germinal center (GC) volume densities measured on spleen H&E images at 36 weeks of age. (**c**) Representative CD3 immunohistochemistry at 36 weeks of age showing increased immune cell infiltrations (arrows) in lung (top), kidney (middle) and liver (bottom) of *nfkb1*^*−/−*^, but not wt, mice. (**d**) Frequencies of CD3-positive cells in livers at the indicated ages. (**e**) Frequencies of CD3-positive cells in livers at 32 weeks of age following treatment of mice with ibuprofen or vehicle for 8 weeks. (**f**) Frequencies of CD3-positive cells in livers at 12 weeks of age following treatment of mice with the antioxidant BHA or vehicle for 4 weeks. (**g**) Representative α smooth muscle cell actin (α-SMA) immunohistochemistry showing increased pro-fibrotic activity in livers from *nfkb1*^*−/−*^ mice. (**h**) Expression of NF-κB target genes in liver (qPCR array) at the indicated ages, *n*=4 per group. All genes that are significantly changed (*P*<0.05) in at least one group are shown. (**i**) Cytokine expression pattern in liver at 36 weeks of age, *n*=4 per group. Green star indicates proteins upregulated in *nfkb1*^*−/−*^ (*P*<0.10, *t*-test), red star indicates that mRNA upregulation (**h**) was confirmed at protein level, black star indicates that mRNA upregulation was not confirmed at protein level.

As *nfkb1*^*−/−*^ mice showed aggravated responses to pro-inflammatory stimuli^20^,^21^, we tested whether these mice would display evidence of sterile, chronic inflammation without any external stimuli. This was in fact the case: *nfkb1*^*−/−*^ mice showed progressive splenomegaly, increasing plasma concentrations of the major pro-inflammatory cytokine IL-6 and increasing infiltration of CD3^+^ immune cells in the liver and other organs (Table 1 and Fig. 1a–d). While levels of these inflammatory markers were mostly inconspicuous at a young age (12–18 weeks), they increased in 36-week-old *nfkb1*^*−/−*^ mice to levels similar to those found in 132-week-old wild-type (wt) controls (Table 1 and Fig. 1a). Detailed analysis of the spleen showed enlargement of both total white pulp and of the B cell-containing germinal centres (Fig. 1b), indicating a humoral immune response. Infiltration of CD3^+^ immune cells into multiple tissues including lung, kidney and liver was increased in *nfkb1*^*−/−*^ mice (Fig. 1c). Kinetic analysis of the liver showed progressive infiltration of CD3^+^ cells with age (Fig. 1d), which could be suppressed by treatment with the anti-inflammatory cyclooxygenase inhibitor ibuprofen (Fig. 1e) or the antioxidant butylated hydroxyanisole (BHA; Fig. 1f) and was accompanied by progressive fibrosis (Fig. 1g). Out of 78 NF-κB target genes, 43 were upregulated in 36-week-old *nfkb1*^*−/−*^ livers and 7 (including *nfkb1*) were downregulated in comparison to wt mice at the same age (Fig. 1h). Even at twice this age (72 weeks), considerably fewer NF-κB target genes (nine up and two down; Fig. 1h) were induced in wt mice. Fifty-one out of 80 tested pro-inflammatory cytokines, chemokines, immunoreceptors and cell adhesion molecules were measurably expressed (by antibody array) in wt and/or *nfkb1*^*−/−*^ livers. Of these, 25 were more abundant (*P*<0.10, *t*-test) in *nfkb1*^*−/−*^ livers, while only 3 tended to be less abundant (Fig. 1i). Upregulation of 10 out of 15 NF-κB target genes from Fig. 1h was confirmed at the protein level (Fig. 1i). Certain pro-inflammatory cytokines, for example, TNF-α, were already upregulated at a very young age (12 weeks) in liver (Supplementary Fig. 1). Together with published results^21^,^22^,^23^ these data show that chronic systemic inflammation in *nfkb1*^*−/−*^ mice progresses with age.

### Accelerated ageing in *nfkb1*^*−/−*^ mice

**Figure 2:**
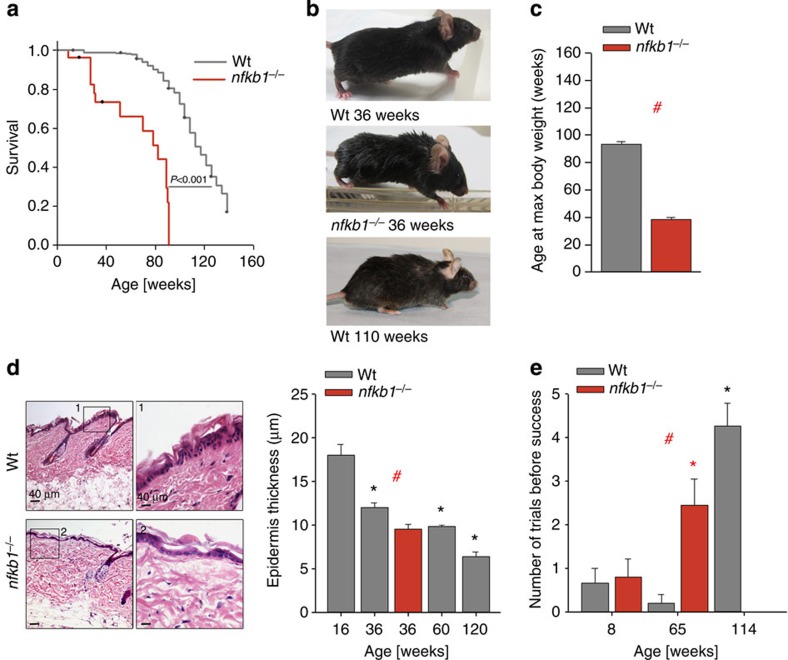
Premature ageing of *nfkb1*^*−/−*^ mice. (**a**) Kaplan–Meier survival curves (right censored) of wt and *nfkb1*^*−/−*^ mice, log-rank test, *n*≥27. (**b**) Representative images of wt mice and an *nfkb1*^*−/−*^ mouse at the indicated ages showing premature hair greying and scruffy fur in *nfkb1*^*−/−*^ mice. (**c**) Age at maximum body weight in *nfkb1*^*−/−*^ and wt mice (*n*=56 for wt, *n*=8 for *nfkb1*^*−/−*^, *P*<0.05, *t*-test). (**d**) Representative skin sections of 36-week-old animals, H&E stain. Boxed areas are shown at higher magnification. Mean epidermal thickness at the indicated ages is shown on the right (*n*≥5). (**e**) Neuromuscular coordination measured as number of unsuccessful attempts to remain on a straight rod for 60 s (*n*=6 to 10 per group). All data are M±s.e.m. Significant differences (ANOVA with *post hoc* Holm–Sidak test, *P*<0.05) to respective controls of the same genotype are indicated by *, and between wt and *nfkb1*^*−/−*^ strains at the same treatment by #.

As chronic inflammation had been linked to ageing^1^,^2^, we investigated whether *nfkb1*^*−/−*^ mice might age faster than their wt counterparts. We tested a wide range of biomarkers of mouse ageing in *nfkb1*^*−/−*^ mice at 36 weeks of age, their C57Bl/6 littermates at the same age and in old C57Bl/6 mice at 104 and 132 weeks (Table 2). With the exception of indicators of muscle weakness (ataxia, sarcopenia and kyphosis) and of spontaneous tumours, all tested biomarkers of ageing were more frequently positive in 36-week-old *nfkb1*^*−/−*^ mice as in their wt counterparts and often reached levels similar to wt mice aged ≥2 years (Table 2). Both mean and maximum lifespan were reduced in *nfkb1*^*−/−*^ mice (Fig. 2a). At ~\n36 weeks of age, adult *nfkb1*^*−/−*^ mice showed frequent hair loss, early hair greying (starting at ~\n20 weeks of age), scruffy fur and cachexia, comparable to wt mice aged 104 weeks or older (Fig. 2b and Table 2). In older *nfkb1^−/−^* mice (ages above 44 weeks) massive hair loss, severe skin inflammation and delayed wound healing together with kyphosis (evidencing muscle loss) was occasionally seen (Supplementary Fig. 2a). Body mass curves in ageing mice show a typical convex curve with a continuous loss of body mass from a maximum achieved at mid-life. *Nfkb1*^*−/−*^ mice reached their maximum body weights already at ~\n36 weeks of age and showed premature body mass loss thereafter (Fig. 2c). Improved maintenance of body fat at mean and advanced age predicts lifespan in mice^24^. Middle-aged *nfkb1*^*−/−*^ mice (36 weeks of age) evidenced loss of fat from inguinal (subcutaneous), epididymal and mesenteric depots (Supplementary Fig. 2b). Thinning of the epidermis is another characteristic age-associated phenotype in mice. In *nfkb1*^*−/−*^ mice at 36 weeks of age, epidermal thickness was decreased to values found in 60-week-old wt mice (Fig. 2d). Finally, *nfkb*1^*−/−*^ mice lost neuromuscular coordination far earlier than wt mice (Fig. 2e). Thus, *nfkb1*^*−/−*^ mice aged prematurely in multiple organ systems.

### Impaired tissue regeneration in *nfkb1* ^*−/−*^ mice

**Figure 3:**
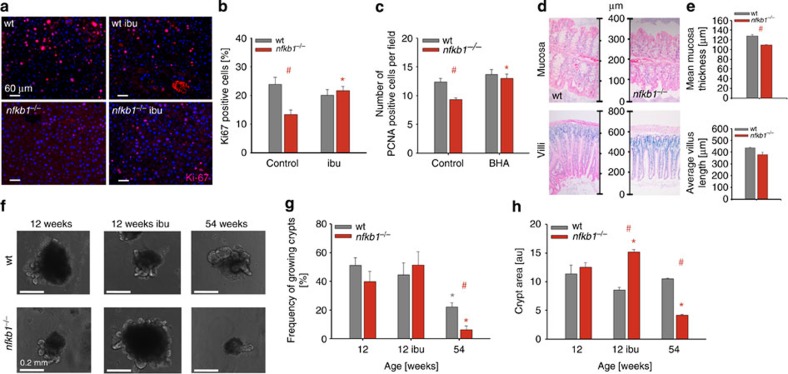
Impaired regeneration in gut and liver of *nfkb1*^*−/−*^ mice is ameliorated by treatment with NSAID. Significant differences (ANOVA with *post hoc* Holm–Sidak test, *P*<0.05) to respective controls of the same genotype are indicated by *, and between wt and *nfkb1*^*−/−*^ strains at the same treatment by #. (**a**) Representative Ki-67 immunofluorescence (red) images from wt (top) and *nfkb1*^*−/−*^ (bottom) livers at 72 h after partial hepatectomy at 12 weeks of age. Mice were pretreated with or without ibuprofen for 4 weeks. (**b**) Frequencies of Ki-67-positive hepatocytes at 72 h after partial hepatectomy in wt and *nfkb1*^*−/−*^ animals. Data are M±s.e.m. from seven animals per group. (**c**) Frequencies of PCNA-positive hepatocytes at 72 h after partial hepatectomy in wt and *nfkb1*^*−/−*^ animals, pretreated or not with the antioxidant BHA for 4 weeks. Data are M±s.e.m. from 7 animals per group. (**d**) Representative colonic (top) and intestinal (bottom) H&E sections from wt and *nfkb1*^*−/−*^ mice aged 36 weeks. (**e**) Quantification of colonic mucosal thickness (top) and villus length (bottom). Data are M±s.e.m. from 30–100 colonic crypts from 4 animals, *P*=0.0001, and from 20–60 villi from 3–5 animals, *P*=0.029. (**f**) Representative images of single crypts isolated from mice at the indicated age and grown for 10 days *in vitro*. (**g**) Frequencies of crypts that grew *in vitro*. (**h**) Crypt size (surface area) at day 10 *in vitro*. Data in (**g**) and (**h**) are M±s.e.m. from 20–200 crypts isolated from three animals per group.

NF-κB-mediated inflammation can stimulate cell proliferation, especially of tumour cells^25^. Conversely, NF-κB is also involved in apoptosis signalling, and NF-κB-driven signalling can stabilize senescent cell arrest *in vitro*^14^,^16^. To understand how NF-kB-driven chronic inflammation can cause accelerated ageing in mice, we first analysed its impact on tissue regeneration. Partial hepatectomy stimulates liver regeneration in wt mice. Hepatocyte proliferation after partial hepatectomy in *nfkb1*^*−/−*^ mice was drastically reduced (Fig. 3a,b) to levels typically found in aged wt mice^26^. However, hepatocyte apoptosis was not enhanced in *nfkb1*^*−/−*^ livers (Supplementary Fig. 3a). Treatment of mice with the anti-inflammatory drug ibuprofen for 1 month before partial hepatectomy completely restored regenerative capacity of hepatocytes in the context of *nfkb1*^*−/−*^ (Fig. 3a,b).

Inflammation is frequently associated with enhanced ROS production and increased oxidative damage^27^. To test whether decreased liver regenerative capacity in *nfkb1*^*−/−*^ was mediated by ROS, mice were treated with the antioxidant BHA before partial hepatectomy. Like ibuprofen, BHA had no effect on hepatocyte proliferation in wt mice, but fully rescued the decreased regenerative potential in *nfkb1*^*−/−*^ livers (Fig. 3c).

The gut epithelium is constantly regenerated from stem cells located at the bottom of the crypts. Low mucosal thickness of the colon and low villus length in the intestine are early morphological indicators of decreasing stem/progenitor function in the ageing gut, leading to malabsorption^28^. Both parameters were decreased in *nfkb1*^*−/−*^ mice aged 36 weeks (Fig. 3d,e) to values similar to those in 52–104-week-old wt mice^28^. This was not associated with enhanced apoptosis (Supplementary Fig. 3b), suggesting a decreased regenerative capacity in either stem or progenitor cells. To address this, we isolated intestinal crypts from 12- and 54-week-old mice and analysed their growth in organotypic culture over a 10-day observation period (Fig. 3f). We measured both the frequencies of crypts that started to grow, which is indicative of stem cell function, and growth rates, indicative of proliferation primarily in the progenitor cell compartment. Crypts isolated from young *nfkb1*^*−/−*^ mice maintained both the ability to grow (Fig. 3g) and growth rates (Fig. 3h) as well as wt crypts. In 54-week-old wt mice (~\n44% of median lifespan), the stem cell-related regenerative potential of intestinal crypts was compromised (Fig. 3g), while progenitor replicative capacity was still intact (Fig. 3h). In 54-week-old *nfkb1*^*−/−*^ mice (64% of median lifespan) both stem and progenitor cell function in the intestinal crypts were decreased (Fig. 3g,h). Ibuprofen treatment had no effect on crypt stem cell function from young mice of either genotype. However, it improved growth rates of crypts from *nfkb1*^*−/−*^ mice (Fig. 3h), suggesting that chronic inflammation in *nfkb1*^*−/−*^ mice limits the proliferative potential of crypt transient amplifying cells in adulthood.

### Chronic inflammation reinforces cellular senescence

As loss of *nfkb1* limited tissue regeneration without enhancing apoptosis and because NF-κB-driven cytokine signalling can reinforce cell senescence *in vitro*^13^,^14^,^16^, we hypothesized that chronic inflammation might aggravate the senescent phenotype and thus limit tissue regeneration.

**Figure 4:**
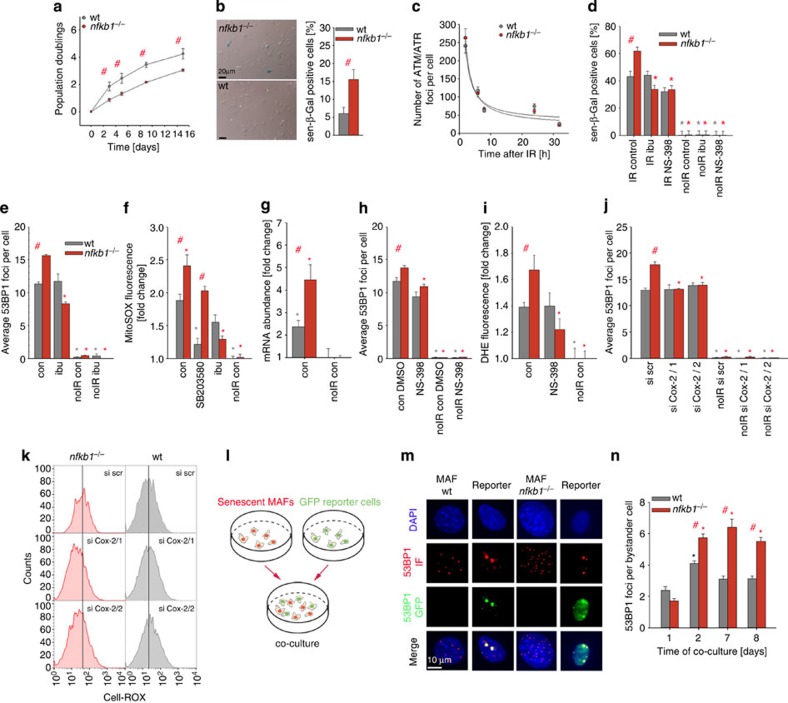
*nfkb1*^*−/−*^ aggravates the senescent phenotype in MAFs. All data are M±s.e.m. from 3or 4 independent strains per condition, if not otherwise indicated. Significant differences (ANOVA with *post-hoc* Holm-Sidak test, *P*<0.05) to respective controls are indicated by *, and between wt and *nfkb1*^*−/−*^ strains at the same treatment/time point by #. (**a**) Growth of *nfkb1*^*−/−*^ and wt MAFs in culture under 21% ambient oxygen. (**b**) Frequencies of sen-β-Gal-positive MAFs after 10 days under 21% ambient oxygen. (**c**) ATM/ATR foci frequencies in wt and *nfkb1*^*−/−*^ MAF nuclei at the indicated times after 10 Gy IR. (**d**) Frequencies of sen-β-Gal-positive MAFs at 3 days after induction of senescence by 10 Gy IR and treatment (or not) with the NSAID ibuprofen (ibu, 0.2 mM) or the COX-2 inhibitor NS-398 (2.5 mM). (**e**) Frequencies of nuclear 53BP1 foci in MAFs at 3 days after 10 Gy IR and treatment (or not) with ibuprofen. (**f**) Mitochondrial superoxide levels measured by MitoSOX fluorescence in MAFs at day 3 after treatment with/without 10 Gy IR and the p38 inhibitor SB203580 (10 μM) or ibuprofen (0.2 mM). (**g**) Expression of the COX-2 gene (normalized to actin) in MAFs 3 days after treatment with/without 10 Gy IR. (**h**) 53BP1 foci frequencies in MAFs treated with the COX-2 inhibitor NS-398 or sham (DMSO) treated. (**i**) Cellular ROS levels (DHE fluorescence intensity) in MAFs after treatment with/without 10 Gy IR and with NS-398. (**j**) 53BP1 foci frequencies in MAFs treated with/without 10Gy IR and either scrambled siRNA or one of two siRNAs against COX-2. (**k**) CELL-ROX fluorescence in MAFs after COX-2 siRNA treatment. One representative experiment out of four per cell type and treatment is shown. (**l**) Scheme of the bystander experiment. wt or *nfkb1*^*−/−*^ MAFs were irradiated to induce senescence and then co-cultured with reporter cells expressing a 53BP1-GFP fusion protein. (**m**) DNA damage foci in senescent MAFs of the indicated genotype (left, shown by 53BP1 immunofluorescence in red) and in co-cultured reporter fibroblasts (right, foci also contain green 53BP1-GFP fusion protein). (**n**) Senescent *nfkb1*^*−/−*^ MAFs induce more DNA damage in non-senescent bystander cells than senescent wt MAFs. 53BP1-GFP foci frequencies in bystander fibroblasts after the indicated times of co-culture are shown.

Mouse fibroblasts senesce spontaneously after few population doublings in 21% ambient oxygen as a consequence of a stress-induced DDR^29^. Mouse adult ear fibroblasts (MAFs) from *nfkb1*^*−/−*^ donors senesced faster than their wt counterparts as indicated by accelerated loss of proliferative capacity (Fig. 4a) and increased expression of the senescence marker senescence-associated β-galactosidase (sen-β-gal; Fig. 4b). To address the underlying mechanisms of accelerated senescence in *nfkb1*^*−/−*^ cells, we used a well-established model of induction of cell senescence by ionizing radiation (IR)^12^,^30^. Radiation-induced DDR can activate NF-κB through an ATM-dependent mechanism^31^. However, the immediate response to DNA damage was not different between *nfkb1*^*−/−*^ and wt MAFs as shown by equal activation of ATM (Fig. 4c and Supplementary Fig. 4a) and p53 (Supplementary Fig. 4a) and equal frequencies of DNA damage foci within 1 day after IR (Supplementary Fig. 4b). Similarly, there was no difference in the abundance of the NF-κB target COX-2 (PTGS-2) immediately after IR (Supplementary Fig. 4a, see also Supplementary Fig. 7).

It is well established that the induction of the senescent phenotype including sen-β-Gal, SASP and enhanced ROS production requires at least 3–7 days following IR^12^,^15^. Multiple feedback loops interconnecting the DDR with SASP and ROS phenotypes via p38MAPK and NF-κB have been described^12^,^13^,^14^,^32^,^33^, all of them stabilizing senescence with some kinetic delay. Accordingly, after a sufficient delay following IR, a wide range of senescence markers was elevated in both wt and *nfkb1*^*−/−*^ MAFs. Importantly, the senescent phenotype was aggravated in *nfkb1*^*−/−*^ cells according to every single marker tested (Fig. 4d-g and Supplementary Fig. 4c–h). Specifically, there were more sen-β-Gal-positive cells (Fig. 4d), more DNA damage foci per nucleus (Fig. 4e), more mitochondrial superoxide was produced per cell (Fig. 4f), more ROS accumulated in the cytoplasm (Supplementary Fig. 4c) and the expression of the CDKN1A and CDKN2A genes encoding the major cyclin-dependent kinase inhibitors p21 and p16 was more strongly upregulated in induced senescence in *nfkb1*^*−/−*^ MAFs (Supplementary Fig. 4d). The SASP was also stronger in *nfkb1^−/−^* MAFs: A cytokine array confirmed enhanced secretion of 36 SASP components in induced senescence in wt MAFs (Supplementary Fig. 4e). Of these, 13 were more abundant in the supernatant of senescent *nfkb1*^*−/−*^ MAFs, while only 6 were less abundant. Moreover, anti-inflammatory cytokines (IL-4 and IL-10) were more robustly downregulated in *nfkb1*^*−/−*^ MAFs (Supplementary Fig. 4e), together indicating an enhanced SASP in senescent *nfkb1*^*−/−*^ fibroblasts. We confirmed upregulation in senescent *nfkb1*^*−/−*^ fibroblasts over wt for the major pro-inflammatory NF-κB targets IL-6 by ELISA (Supplementary Fig. 4f) and TNF-α by qRT–PCR (Supplementary Fig. 4g). In accordance with all other markers, ATM/ATR foci were also more frequent in *nfkb1*^*−/−*^ MAFs at 10 days after IR (Supplementary Fig. 4h).

To understand the mechanisms by which chronic pro-inflammatory signals increased senescence-associated mitochondrial ROS production we first tested the role of p38MAPK. Signalling through p38MAPK drives enhanced mitochondrial ROS^12^ and activates NF-κB^17^ in senescence. Accordingly, inhibition of p38MAPK using SB203580 effectively reduced ROS production in senescent wt MAFs but was much less effective in senescent *nfkb1*^*−/−*^ cells in which NF-κB is constitutively activated independent of p38MAPK (Fig. 4f). In contrast, treatment with the NSAID ibuprofen, which acts preferentially by inhibition of prostaglandin synthesis driven by cyclooxygenases COX-1 and COX-2 (ref. 34), had no effect in wt MAFs but efficiently reduced frequencies of sen-β-Gal-positive cells (Fig. 4d), DNA damage foci frequencies (Fig. 4e) and mitochondrial superoxide (Fig. 4f) in senescent *nfkb1*^*−/−*^ MAFs. COX-2 expression was upregulated in senescence, more so in *nfkb1*^*−/−*^ MAFs than in wt cells (Fig. 4g). Prostaglandin E2, which is synthesized by COX-2, increases in senescence and can induce and maintain multiple markers of the senescent phenotype^35^. Inhibition of COX-2 by the specific inhibitor NS-398 mimicked the effect of ibuprofen by reducing sen-β-Gal-positive cells (Fig. 4d), the DDR (Fig. 4h and Supplementary Fig. 4h) and cellular ROS levels (Fig. 4i) specifically in *nfkb1*^*−/−*^ MAFs. Accordingly, knockdown of COX-2 by two separate siRNAs (Supplementary Fig. 4i) reduced DNA damage foci frequencies (Fig. 4j) and ROS levels (Fig. 4k) exclusively in *nfkb1*^*−/−*^ MAFs. A similar reduction of the DDR was obtained by treating *nfkb1*^*−/−*^, but not wt, MAFs with the antioxidant NAC (Supplementary Fig. 4j). Together, these data show that loss of *nfkb1* stabilizes cell senescence by aggravating mitochondrial ROS production via COX-2 activation and, consequently, enhancing nuclear DDR.

Senescence is not a cell-autonomous process. By secreting bioactive molecules including interleukins, chemokines and ROS^12^,^15^, senescent cells induce powerful bystander effects that spread senescence to neighbouring normal cells^36^,^37^. Given that both ROS and SASP signals are stronger in *nfkb1*^*−/−*^ cells, we hypothesized that these cells, when senescent, might exert a stronger bystander effect. We tested this assumption by co-culture of *nfkb1*^*−/−*^ fibroblasts with reporter cells in which a GFP-53BP1 fusion protein allowed kinetic monitoring of the DDR specifically in bystander cells^36^ (Fig. 4l). As expected, senescent *nfkb1*^*−/−*^ MAFs induced more DNA damage in bystander cells than senescent wt MAFs (Fig. 4m,n). This shows that loss of *nfkb1* reinforces cellular senescence by both autocrine and paracrine signalling. We therefore expected faster accumulation of senescent cells in tissues from *nfkb1*^*−/−*^ mice.

### Feedback between inflammation and telomere dysfunction *in vivo*

**Figure 5:**
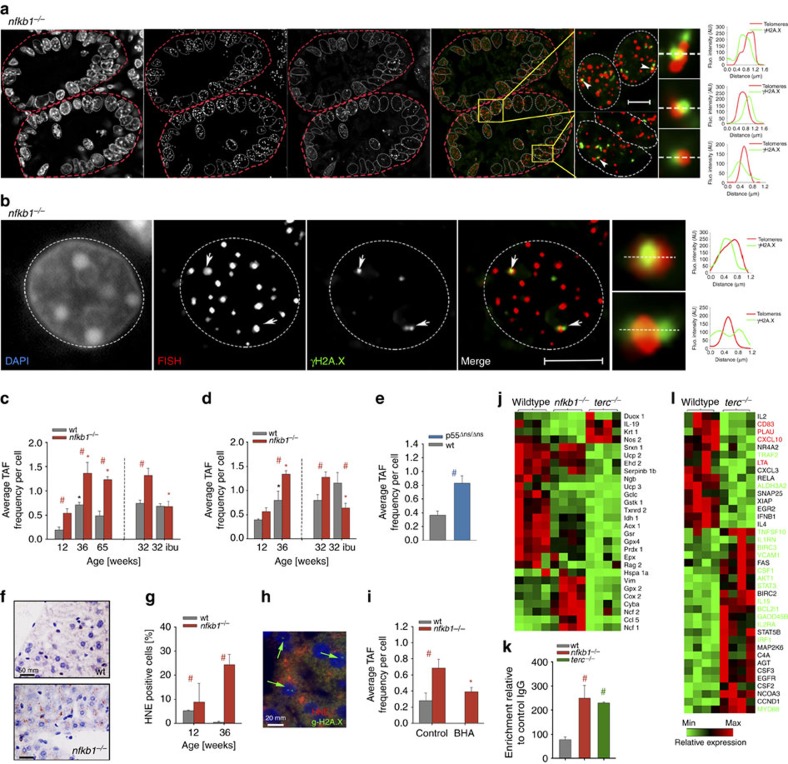
Inflammation causes telomere dysfunction and senescence *in vivo*. All data are M±s.e.m. from 5-6 animals per group. Significant differences (ANOVA with *post-hoc* Holm-Sidak test, *P*<0.05) to control (young) animals of the same genotype are indicated by *, and between wt and transgenic animals at the same age/treatment by #. (**a**) Representative immunoFISH image from a crypt cross-section (*nfkb1*^*−/−*^, 36 weeks). (**b**) Representative immunoFISH image from a *nfkb1*^*−/−*^ hepatocyte nucleus at 36 weeks of age. In (**a**) and (**b**), images are maximum intensity projections of at least 60 planes. Amplified images on the right are from single Z planes where colocalization was found. Graphs represent quantification of γH2A.X and telomere signals in selected regions of interest (dotted lines). Scale bar: 10 μm. (**c**) Frequencies of TAF in hepatocytes from *nfkb1*^*−/−*^ and wt livers at the indicated ages. 32 weeks old animals received either 8 weeks ibuprofen or sham treatment. (**d**) Frequencies of TAF in *nfkb1*^*−/−*^ and wt intestinal crypt enterocytes at the indicated ages. 32 weeks old animals received either 8 weeks ibuprofen or sham treatment. (**e**) TAF frequencies in livers from p55^Δns/Δns^ mice at 20 weeks of age. (**f**) Representative 4-HNE immunohistochemistry in wt (top) and *nfkb1*^*−/−*^ (bottom) liver (centrilobular area). (**g**) Frequencies of 4-HNE-positive hepatocytes at the indicated ages. (**h**) Representative double immunofluorescence image of *nfkb1*^*−/−*^ liver. green: γH2AX, red: 4-HNE, blue: DAPI. Cells that are both positive for γH2AX^+^ and 4-HNE^+^ are marked by arrows. (**i**) Frequencies of TAF in hepatocytes from *nfkb1*^*−/−*^ and wt livers at 12 weeks of age. Animals received either 4 weeks BHA or sham treatment. (**j**) Expression of oxidative stress-related genes in liver (qPCR array) of wt, *nfkb1*^*−/−*^ and late generation *terc*^*−/−*^ mice, *n*=4 per group. All genes that are significantly changed (*P*<0.05) in at least one group are shown. (**k**) NF-κB activity in wt, *nfkb1*^*−/−*^ and late generation *terc*^*−/−*^ liver tissue. (**l**) Expression of NF-κB target genes in wt and late generation *terc*^*−/−*^ liver (qPCR array). *n*=4 per group. All genes that are significantly changed (*P*<0.05) in at least one group are shown. Gene names in green: expression changed in the same direction in *nfkb1*^*−/−*^ and *terc*^*−/−*^ livers. Gene names in red: expression changed in opposite direction (comp. Fig. 1h).

Telomere dysfunction is an important driver of cell senescence^38^, and TAF (Fig. 5a,b) are established as markers of telomere dysfunction and cell senescence in human and mouse tissues^9^,^11^,^39^,^40^,^41^. TAF frequencies increased with age in liver hepatocytes and in enterocytes in intestinal crypts from wt mice (Fig. 5c,d) similar to other markers of cell senescence^11^,^42^,^43^. TAF frequencies were always higher in *nfkb1*^*−/−*^ mice (Fig. 5c,d), reaching levels similar to those found in late-generation *terc*^*−/−*^ mice (Supplementary Fig. 5a), a well-established model for telomere dysfunction-driven senescence^10^,^12^. As expected for dysfunctional telomeres, chromatin bridges were observed between hepatocyte nuclei in *nfkb1*^*−/−*^ livers following partial hepatectomy (Supplementary Fig. 5b). To test whether the increase of TAF-positive cells was caused by inflammation rather than some other effect of p105/p50 deficiency, we first treated *nfkb1*^*−/−*^ mice for 8 weeks with the NSAID ibuprofen. Ibuprofen has complex, context-dependent effects on expression of pro-inflammatory cytokines^44^,^45^, but robustly suppresses systemic COX activity^34^. Enhanced TAF frequencies in *nfkb1*^*−/−*^ tissues were completely prevented by this treatment (Fig. 5c,d). To further verify the causal role of inflammation for induction of telomere dysfunction *in vivo*, we measured TAF frequencies in livers from an independent transgenic model of chronic inflammation. p55^Δns^ knock-in mice express a mutated TNFR1 ectodomain that is incapable of shedding, leading to chronic activation of TNF-α signalling and chronic low-grade inflammation specifically in the liver^46^. As this phenotype is confined to the liver^46^, it did not cause obvious progeria in the mice. However, p55^Δns/Δns^ livers showed hepatocyte TAF frequencies higher than in wt and similar to those in *nfkb1*^*−/−*^ livers (Fig. 5e), and mRNA expression of the senescence marker CDKN2A (p16) was increased in p55^Δns/Δns^ livers (Supplementary Fig. 5c). Together, these data show that telomere dysfunctional cells accumulate in different mouse models of chronic inflammation.

Telomere dysfunction in *nfkb1*^*−/−*^ mice was not associated with shortened telomere length (Supplementary Fig. 5d). Moreover, telomerase interacts with NF-κB in a positive feedback loop^47^ and therefore tended to be downregulated under ibuprofen in *nfkb1*^*−/−*^ mice (Supplementary Fig. 5e), excluding improved protection of telomeres by telomerase as a cause of ibuprofen-mediated rescue of telomere dysfunction.

However, telomeres are exquisitely sensitive to damage by ROS, generated extrinsically^39^ or intrinsically^11^,^48^ as a by-product of normal cellular metabolism. In cell senescence, ROS production is enhanced, generating DNA damage and increasing the DDR in a positive feed-forward loop^12^. This loop is aggravated by telomere dysfunction, as seen in late generation *terc*^*−/−*^ mice^12^,^49^ and in *nfkb1*^*−/−*^ cell senescence *in vitro* (Fig. 4). Accordingly, oxidative stress as measured by accumulation of 4-HNE, a marker for lipid peroxidation, was increased in *nfkb1*^*−/−*^ livers (Fig. 5f,g) as well as broadband autofluorescence, another marker of oxidative damage (Supplementary Fig. 5f). 4-HNE-positive hepatocytes in *nfkb1*^*−/−*^ livers were also positive for the DNA damage- and senescence marker γH2AX (Fig. 5h). Importantly, treatment of mice for 4 weeks with the antioxidant BHA rescued the TAF increase mediated by knockout of *nfkb1* (Fig. 5i). Additional markers of cell senescence including frequencies of γH2AX^+^ PCNA^−^ cells^42^ (Supplementary Fig. 5g), frequencies of ATM/ATR-positive cells (Supplementary Fig. 5h) and nuclear size (Supplementary Fig. 5i) were also enhanced in hepatocytes from ageing *nfkb1*^*−/−*^ mice. Moreover, there was a strong preference of 4-HNE-positive hepatocytes to form clusters in *nfkb1*^*−/−*^ livers (Supplementary Fig. 5j,k) as expected if a bystander effect contributed to senescent cell accumulation^36^. Together, these data show that, like *nfkb1*^*−/−*^ fibroblasts *in vitro*, *nfkb1*^*−/−*^ hepatocytes *in vivo* are more prone to senesce, driven by positive feedback between ROS production and DDR and involving telomere dysfunction. This was not restricted to the liver; frequencies of γH2AX^+^ PCNA^−^ cells were similarly enhanced in intestinal crypts (Supplementary Fig. 5l). In intestinal crypts, both TAF analysis (Supplementary Fig. 5m) and γH2AX/PCNA immunofluorescence (not shown) localized senescent enterocytes preferentially around the position of the transient amplifying cells, suggesting preferential exhaustion of progenitor cells. Cells with an activated DDR were also more abundant in other tissues from *nfkb1*^*−/−*^ mice including heart, colon and spleen (not shown).

As expected, COX-2 was upregulated in *nfkb1*^*−/−*^ livers while major antioxidant enzymes including GPX4, Prdx1, GSTK1, Txnrd2 and the Nrf2 transcriptional targets AOX1 and GCLC were downregulated (Fig. 5j). As in MAFs, COX-2 upregulation is specific to *nfkb1*^*−/−*^ mice, while antioxidant downregulation is shared in livers from late-generation *terc*^*−/−*^ mice (Fig. 5j), in which increased cellular ROS production is caused by a DDR triggered by dysfunctional telomeres^12^,^49^.

Having shown enhanced telomere dysfunction and senescence in two different models of chronic inflammation *in vivo*, we investigated whether conversely telomere dysfunction would lead to a pro-inflammatory state, for example, in tissues from late-generation *terc*^*−/−*^ mice. This was in fact the case: NF-κB activity, measured as the amount of TNF-α promoter DNA precipitated using an anti-p65 antibody, was equally enhanced in *nfkb1*^*−/−*^ and late-generation *terc*^*−/−*^ livers (Fig. 5k). Moreover, out of 78 tested NF-κB target genes, the expression of 39 genes was significantly changed in late-generation *terc*^*−/−*^ livers, with the majority (24 genes) upregulated in *terc*^*−/−*^ (Fig. 4l). The overlap in NF-κB target gene regulation between *terc*^*−/−*^ and *nfkb1*^*−/−*^ livers was considerable: 15 genes were equally regulated in *terc*^*−/−*^ and *nfkb1*^*−/−*^, while only 4 genes were changed in the opposite direction in both models (compare Figs 1h and 5l).

Together these data suggest that there exists a positive feedback loop system between telomere dysfunction, senescence-associated ROS production and pro-inflammatory signalling that induces and stabilizes senescence *in vivo*, which in turn limits regenerative capacity of tissues.

### Frequencies of senescent cells in sensitive tissues predict lifespan

**Figure 6:**
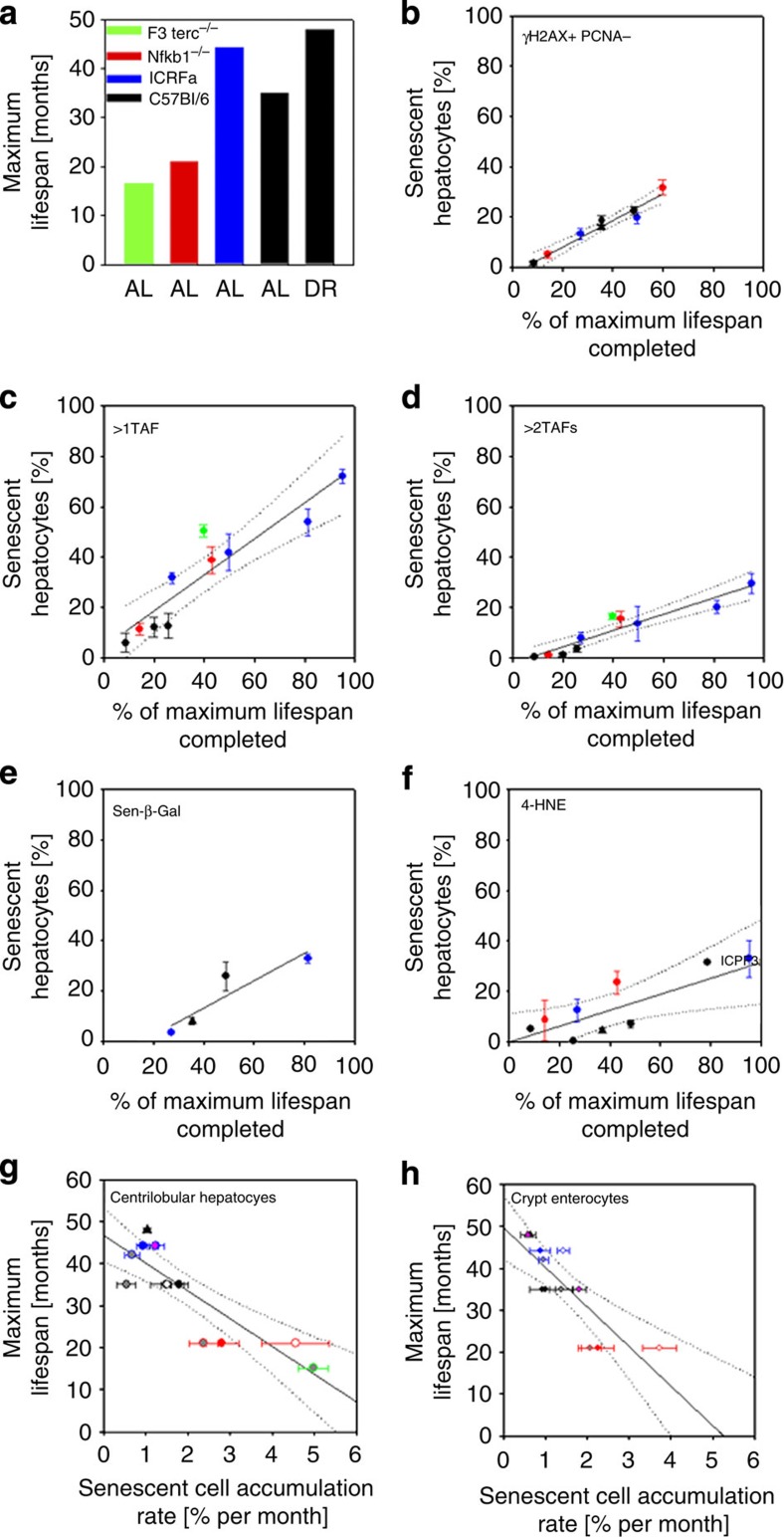
Accumulation of senescent cells predicts lifespan in mice over a wide range of interventions. (**a**) Maximum lifespan of males in different cohorts of mice under AL feeding and dietary restriction (DR). See Methods for cohort description. (**b**–**f**) Frequencies of senescent centrilobular hepatocytes measured as γH2AX^+^ PCNA^−^ cells (**b**), cells with >1 TAF (**c**), with >2TAFs (**d**), Sen-β-Gal positive cells (**e**) or 4-HNE- positive cells (**f**) versus relative age (calculated as % of maximum lifespan completed). Symbol colours indicate the same strains as in (**a**), triangle indicates a strain under dietary restriction. Data are M±s.e.m. from at least 3 mice per age group. (**g**) Maximum lifespan per cohort versus rate of accumulation of senescent hepatocytes, calculated by linear regression of senescent cell frequencies against age. Symbol outlines indicate strain/condition as before. Symbol fills indicate the senescence marker used: outline colour: γ-H2AX^+^ PCNA^−^, pink: sen-β-Gal, white: >1TAF, grey: >2TAFs. (**h**) Maximum lifespan versus rate of accumulation of senescent crypt enterocytes. Symbols as before.

Continuous regeneration is an essential feature of life. If telomere dysfunction and associated cell senescence is a major limitation to tissue regeneration one should expect that accumulation of senescent cells might quantitatively predict lifespan in mice. To test this assumption we used cohorts of mice that differed almost threefold in their maximum (Fig. 6a) and median (Supplementary Fig. 6a) lifespan while being kept under identical housing conditions in our dedicated ageing mice unit. Lifespan differences were due to either genetic (*nfkb1*^*−/−*^, late-generation *terc*^*−/−*^) or environmental (dietary restriction) intervention or to selected breeding (ICRFa). Senescent cell frequencies in crypt enterocytes and centrilobular hepatocytes were measured at different ages using multiple markers. We counted γ-H2AX^+^PCNA^−^ cells, TAF^+^ cells (separated into cells with >1TAF and with >2TAFs), sen-β-Gal^+^ cells and (in liver only) 4-HNE^+^ cells as markers of senescence. Surprisingly, senescent cell frequencies over all disparate ageing models fitted well into the same linear correlation with relative age, calculated as the percentage of maximum lifespan of the strain (Fig. 6b–f and Supplementary Fig. 6b). Similarly strong correlations were found if age was calculated as percentage of median lifespan (Supplementary Fig. 6c,d). A comparison between the different markers showed that >1TAF and >2TAF data flanked the γ-H2AX^+^PCNA^−^, Sen-β-Gal^+^ and 4-HNE^+^ estimates on both sides, indicating that the minimum number of TAF associated with cell senescence is between 2 and 3 in both hepatocytes and enterocytes. 4-HNE, measuring a specific lipid peroxidation product, is arguably the most indirect marker of senescence, which might explain why it showed the largest variation between mouse models.

To assess the strength of the quantitative association between senescent cell accumulation and lifespan, we calculated accumulation rates for senescent cells over time separately for each of the mouse models and each marker. These data linearly predict maximum (Fig. 6g,h) and median lifespan (Supplementary Fig. 6e,f). Interestingly, quantitative predictions are very similar for liver and gut. Whether this indicates that there is an upper frequency of senescent cells that can be tolerated in any tissue compartment awaits further examination.

## Discussion

Tissue repair and regeneration are of prime importance for the maintenance of tissue homeostasis during ageing. They are dependent on maintaining functional capacity in tissue-specific stem and progenitor cells, but this functionality is known to decrease with ageing in multiple tissues, caused at least partially by activation of DNA damage checkpoints^50^,^51^,^52^. As exemplified by repair of infected or otherwise damaged tissue, inflammation is often an essential component of tissue regeneration. Here we suggest that failure to resolve the former impairs the latter because inflammation causes DNA damage and, especially, telomere dysfunction, which is a potent activator of persistent DNA damage checkpoint activity. Pro-inflammatory signals can cause telomere dysfunction because they are closely integrated in multiple positive feedback loops with stress and nutrient signalling pathways (involving p38MAPK, TGF-β, mTOR and others) that contribute to control of mitochondrial function and ROS production^12^,^13^,^14^,^17^. Specifically, our data show a major role for the NF-κB target COX-2 in instigating oxidative stress, which in turn contributes to induction and maintenance of a DDR. Telomeres are preferential sites for DNA damage accumulation^11^,^39^, because they are deficient for various types of DNA repair^53^,^54^. Thus, inflammation acting chronically *in vivo* aggravates telomere dysfunction by increasing oxidative stress at least partially through COX-2 activation. This then accelerates accumulation of senescent cells, which intensifies pro-inflammatory and pro-oxidant signalling by the SASP response^13^,^32^ and by induction of mitochondrial dysfunction^55^, spreading DNA damage and senescence towards bystander cells^36^,^37^. Interestingly, we found a pro-inflammatory phenotype in *nfkb1*^*−/−*^ cells *in vitro* (in terms of secreted cytokines, COX-2 expression and ROS production) only after induction of senescence (Fig. 4). Together with the increased frequencies of senescent cells in *nfkb1^−/−^* tissues (Figs 5 and 6) this suggests that aggravated cell senescence could at least partly be instrumental for the establishment of ‘classical’ inflammatory phenotypes like immune cell infiltration into solid organs. Both recent intervention studies^18^ and our correlative data presented here strongly underscore the importance of cell senescence for determination of ageing rate and lifespan in mammals. Tissue resident stem cells^51^,^52^ and fast proliferating progenitors might be most sensitive to the consequences of DNA damage checkpoint activation and thus organ repair becomes increasingly suboptimal with ageing.

A limitation of our study is that we did not attempt to rescue lifespan in *nfkb1*^*−/−*^ mice by anti-inflammatory treatment because of the known long-term side effects of NSAIDs like ibuprofen^34^. However, medium-term (1–2 months) treatment of mice with either ibuprofen or the antioxidant BHA rescued telomere dysfunction and regenerative capacity in the *nfkb1*^*−/−*^ background. Moreover, long-term treatment with the NSAID aspirin prolonged lifespan in genetically heterogeneous wt mice^56^.

Taken together, our data suggest that loss of regenerative potential and accelerated ageing in *nfkb1*^*−/−*^ mice are due to chronic activation of the NF-κB–COX-2–ROS axis causing accelerated and aggravated cell senescence in multiple tissues. NF-κB activity, COX-2 expression and ROS levels are also elevated in late generation *terc*^*−/−*^ mice, in which progeria is driven by telomere dysfunction causing apoptosis and senescence. This indicates that pro-inflammatory signals, ROS, persistent DDR and senescence are interconnected in positive feedback loop(s) not only *in vitro*^12^,^13^,^14^,^17^ but also *in vivo*. The fact that senescent cell accumulation quantitatively predicts lifespan not only for progeroid but also for long-lived mouse models suggests that feedback between inflammation, ROS and cell senescence might be a general determinant of the rate of ageing in mammals.

Our findings may have implications for human ageing in the context of many, if not all, of the modern chronic disease states. Type II diabetes, liver disease, arthritis, CVD, metabolic syndrome or dementia, to name just a few, have in common that ageing is the major risk factor. Moreover, they are associated with chronic low-grade inflammation (or ‘parainflammation’) and, possibly, with cell senescence^57^. Our data suggest that once systemic inflammation is established, for example, in consequence of any single disease, ageing in multiple organ systems might accelerate, and this would increase the risk for multimorbidity. There is epidemiological evidence that prevalence of a chronic disease is a significant risk factor for incidence of additional, often multiple age-associated degenerative diseases^58^. Multimorbidity, frailty and mortality in the elderly are all associated with markers of chronic systemic inflammation^59^,^60^. Our data suggest that long-term anti-inflammatory intervention in elderly patients with age-associated degenerative disease might reduce steady-state levels of senescent cells, which could have preventive potential. Finally, our data show that mouse models for chronic inflammation like the *nfkb1*^*−/−*^ mouse provide a model for further interrogating these mechanisms and testing the ‘anti-ageing’ potential for interventions such as anti-inflammatory drugs or lifestyle modifications that have an impact on the inflammatory process.

## Methods

### Animals

Experiments were performed on male *nfkb1*^*−/−*^ mice on a pure C57Bl/6 background and C57Bl/6 wt controls. Pure background mice were a gift from Jorge Caamano, (Birmingham University, UK). Experiments were performed at 36 weeks of age if not indicated otherwise. Immunohistochemistry was also performed on mixed C57Bl/6;129PF2/J *nfkb1*^*−/−*^ and F2 hybrid *nfkb1*^+/+^ wt control mice (Jackson Laboratories, Bar Harbor, ME, USA) with identical results. p55^Δns/Δns^ mice and their wt littermate controls have been described^46^. Male late-generation (F3–F4) *terc*^*−/−*^ mice were bred from B6/Cg-TERC^tm1Rdp^/J (Jackson Laboratories). Lifespan studies were also performed in male C57Bl/6 under *ad libitum* feeding (AL) and under dietary restriction (DR, 60% of AL intake)^61^ and in male ICRFa (a long-lived substrain of C57Bl/6)^62^. Mice were housed in cages (56 cm × 38 cm × 18 cm, North Kent Plastics, Kent, UK) of groups of 4–6 that did not change from weaning. Mice were provided with saw dust and paper bedding and had AL access to water. Mice were housed at 20±2 °C under a 12 h light/12 h dark photoperiod with lights on at 07:00 hours. Group sizes for lifespan experiments were (censored events in brackets): *nfkb1*^*−/−*^ 27 (18), C57Bl/6 AL 310 (172), C57Bl/6 DR 241 (157), IRCFa 2391 (1,061) and F3TERC^*−/−*^ 13 (0). Maximum lifespan was estimated as the lifespan of the longest living 1–3% of the cohort. Medians were calculated from right-censored Kaplan–Meier curves. Ethical approval was granted by the LERC Newcastle University, UK. All experiments were undertaken in compliance with UK Home Office legislation under the Animals (Scientific Procedures) Act 1986. Organs were either fixed in 4% paraformaldehyde or snap-frozen in liquid nitrogen and stored at −80 °C. Telomerase activity was measured by TeloTAGGG Telomerase PCR ELISA kit (Roche). Fixed tissues were processed and embedded in paraffin. All sections were cut at a thickness of 3 μm.

70% partial hepatectomy (PHX) was performed in 12–week-old mice according to the method of Higgins and Anderson^63^.

### Ibuprofen and BHA treatment

For liver and gut regeneration studies, mice were given ibuprofen mixed in their food to a daily dosage of 50 mg per kg (mouse) per day. Treatment started at 8 weeks of age for 4 consecutive weeks. For senescence studies, mice received ibuprofen via pump (mini-osmotic pump, Alzet, model 2004) for a period of 8 weeks (starting at 24 weeks of age). Ibuprofen was dissolved in PEG and DMSO (50:50) to a daily dosage of 50 mg per kg. A small incision was made on the right flank and a mini pump was inserted subcutaneously and the wound was repaired with 7 mm clips. After 28 days a replacement was implanted. Under general anaesthesia, pumps were surgically removed and a wound repair was performed. A small incision was made on the left flank and a new mini pump was inserted subcutaneously and the wound was repaired with 7 mm clips. 8-week-old wt or *nfkb1*^*−/−*^ mice were fed BHA (0.7% w/w) or normal chow for 4 weeks before undergoing partial hepatectomy.

### Neuromuscular coordination

The tightrope test is a widely used and extensively validated marker of ageing and shows differences in neuromuscular coordination^64^. The mice were placed on a horizontal bar with a diameter of 1.5 cm and a length of 60 cm. The time spent on top of the bar was recorded. A trial was considered successful if the animal could remain on the bar for 60 s without falling off. Each mouse was given five consecutive trials.

### Cell culture

Ear clippings were transported and stored (not longer than 1 h) in DMEM containing serum on ice. Punches were washed three times with serum-free media, finely cut and incubated for 2–3 h at 37 °C in 2 mg ml^−1^ collagenase A in DMEM. A single-cell suspension was obtained by repeated pipetting and passing through a 24G fine needle. Cells were centrifuged for 10 min at 1,000 r.p.m. and cultured in Advanced DMEM/F-12 (DMEM, Invitrogen) plus 10% fetal calf serum (Sigma) in 3% O_2_ and 5% CO_2_. Each cell strain was derived from a separate donor. MAFs were seeded and allowed to grow for 24 h, then X-ray-irradiated with 10 Gy using a PXI X-Rad 225 (RPS Services Ltd). Immediately following irradiation, media was replaced.

Cells were treated with 10 nM siRNA (Qiagen, SI101392391, SI01392398 or no. 1027281) at 1 day before IR in HiPerFect (Qiagen). Ibuprofen (Sigma, no. I7905-1G, 0.2 mM) was added to the cell culture media immediately after IR. Inhibitors were used from immediately after IR at the following concentrations: SB203580 at 10 μM, NS-398 at 2.5 mM. To measure mitochondrial superoxide^55^, cells were stained in 5 μM MitoSOX Red (Molecular Probes; http://www.invitrogen.com) for 10 min at 37 °C, and FL3 median fluorescence intensity was measured by flow cytometry (Partec PAS; http://www.partec.com). Specificity of MitoSOX for superoxide has been shown by the manufacturer, and its mitochondrial localization was tested by co-staining with Mitotracker Green. Cellular peroxide levels were assessed by staining with 5 μM DHE (Molecular Probes) for 10 min in the dark at 37 °C and analysis of FL3 fluorescence. Cellular ROS levels were assessed by staining with 25 μM CellROX Orange (Life Technologies) for 30 min in the dark at 37 °C and analysis of FL2 fluorescence. The flow cytometer was calibrated using fluorescent microspheres. All data are mean±s.e.m. from at least three independent experiments with measurements in duplicate and 10^4^ cells per measurement.

### Bystander experiments

A total of 10,000 MAFs from wt and *nfkb1*^*−/−*^ mice were X-ray-irradiated with 10 Gy. At the indicated time points, media was changed and 10,000 human reporter cells (transduced with the AcGFP–53BP1c reporter^65^) were added and co-cultured for 1 or 2 days. Cells were fixed in 2% paraformaldehyde and stored at −80 °C until staining was performed.

### Quantitative analysis of cluster probability

A total of 10–20 images per section were taken using a × 20, 0.5NA objective and tiled together. The total number of cells (*N*) and the number of 4-HNE-positive cells (*M*) per tiled image were counted as well as the total number of neighbours (*n*_*i*_) and the number of 4HNE-positive neighbours (*m*_*i*_) for each of the positive cells (*i*=1...*M*). To test whether a positive cell was more likely to have positive neighbours than negative neighbours given the prevalence of positive cells in the image (that is, whether positive cells were clustered to a larger degree than expected by random chance), a hyper-geometric test for over-representation of positive cells in the neighbourhood of each positive cell was carried out. A *P*-value (*p*_*i*_) for over-representation was calculated for each neighbourhood. The probabilities *p*_*i,..,M*_ were corrected for multiple comparisons by false discovery rate control to give *q*-values (*q*_*i,...,M*_). The fraction (*F*) of positive cells having significantly more positive neighbours than expected by chance given the background proportion of positive cells observed was estimated as^36^:


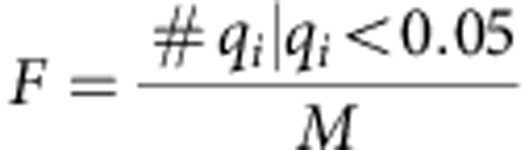


### ELISA

Cells were grown in 75 cm^2^ flasks. Two days after irradiation, medium was replaced by fresh serum-free medium. After 24 h, media was collected, sterile-filtered (0.4 μm pore size) and frozen at −80 °C. Cells were trypsinized, counted and pelleted for protein concentration assay. ELISAs (Murine IL-6 ELISA Mini Kit, PeproTech, no. 900-M50; murine TNF-α ELISA Mini Kit, PeproTech, no. 900-M54) were performed according to the manufacturer’s instructions.

### Quantibody array

Wt and *nfkb1*^*−/−*^ mouse ear fibroblast (isolated from four different mice each) were seeded in 75 cm^2^ flasks. Media was changed 8 days after IR to 4 ml serum-free media and collected after 2 days. Supernatant was centrifuged for 10 min at 2,000 r.p.m. at 4 °C. The supernatant (3 ml) was stored at −80 °C for analysis. Frozen livers from 12- and 36-week-old wt and *nfkb1*^*−/−*^ mice were pulverized in a liquid nitrogen-cooled mortar and pestle. Tissues were lysed using 0.5% Triton-100 in TRIS (50 mM, pH 7.4) and 1 tablet of proteinase inhibitor complete (Roche, no. 05892953001). Lysis buffer (700 μl) was added and lysate was placed in a Qiagen shredder and centrifuged at full speed for 2 min followed by another spin for 10 min at 5,000 r.p.m. at 4 °C. Supernatant was transferred in several tubes, protein concentration was measured and samples were stored at −80 °C. Analysis was performed by RayBiotech Inc. (Insight Biotechnology Ltd, Wembley UK). For data analysis proteins were included if they showed expression above background in at least two of the conditions. Using Gene Cluster 3.0, data from the four replicates were averaged and normalized. Results were visualized in Java TreeView.

### Crosslinked chromatin immunoprecipitation assay

ChIP assay was carried out using crosslinked chromatin prepared from wt, *terc*^*−/−*^ and *nfkb1−/−* livers. Briefly, the chromatin was prepared by resuspending powdered, frozen liver in 10 ml cold PBS with protease inhibitors and crosslinked with 1% formaldehyde for 5 min. The reaction was stopped by adding 0.125 M glycine, cells spun and pellet resuspended in lysis buffer (1% SDS, 10 mM EDTA, 50 mM Tris–HCl (pH 8.1)) for 20 min before sonication for 10 min (10 cycles of 30 s on, 30 s off) in Diagenode Bioruptor. The sonicated material was spun down and chromatin-containing supernatant was collected. Then, 25 μg of chromatin was incubated with 5 μg of anti-p65 antibody (sc109x, Santa Cruz) or irrelevant control antibody (ab46540, Abcam) overnight at 4° C. The complexes were collected using 50 μl of blocked Staph A membranes, which were then serially washed, complexes eluted off and crosslinks reversed. Genomic DNA was purified and used in quantitative PCR with primers that span the region of interest. Each PCR reaction was performed in triplicate and the analysis was repeated at least three times from independent ChIP experiments. A signal intensity value for each sample was calculated from the average of the experiments. Average values of eluates were normalized to average values of control antibody sample and expressed as fold enrichment above background (that is, control antibody). Quantitative PCR amplification was carried out using primers mTNFa chip F1 5′-ACACACACACCCTCCTGATT-3′ and mTNF-α chip R1 5′-GCGGGGAAAAGCTCTCATC-3′.

### mRNA expression analysis

RNA was isolated from frozen tissue using RNeasy Mini Kit and QIAshredder. RNA quality was checked on the Bioanalyzer. Total RNA was reverse-transcribed using Omniscript Reverse Transcription Kit (Qiagen). QPCR was run in triplicates using the following primers:

p21 forward 5′-TGCCAGCAGAATAAAAGGTG-3′

p21 reverse 5′-TTGCTCCTGTGCGGAAC-3′

TNF α forward 5′-ATGCGTCCAGCTGACTAAA-3′

TNF α reverse 5′-TCCCCTTCATCTTCCTCCTT-3′

β-Actin forward 5′-TAAGGCCAACCGTGAAAAG-3′

β-Actin reverse 5′-ACCAGAGGCATACAGGGACA-3′

Cox-2—Qiagen (Mm_Ptgs2_1_SG QuantiTect primer assay, order no.: QT00165347)

### qPCR array

Eighty four NF-κB target genes and oxidative stress response genes were analysed using the RT^2^ Profiler PCR Array Format D (no. PAMM-225Z and no. PAMM-065Z Qiagen) following the manufacturer’s recommendations.

### Histochemistry and immunofluorescence

Paraffin sections were deparaffinized with Histoclear and ethanol, antigen was retrieved by incubation in 0.01 M citrate buffer (pH 6.0) at 95 °C for 20 min. Slides were incubated in 0.9% H_2_O_2_ for 30 min and afterwards placed in blocking buffer (Rabbit IgG, no. PK-6101; Vector Lab) for 30–60 min at room temperature. Livers were further blocked with Avidin/Biotin (Vector Lab, no. SP-2001) for 15 min each. MAFs were washed briefly with PBS and fixed for 10 min with 2% paraformaldehyde dissolved in PBS. Cells were permeabilized for 45 min with PBG. Primary antibodies were applied overnight at 4 °C. Slides were washed three times with PBS and incubated for 30 min with secondary antibody (no. PK-6101; Vector Lab). Antibodies were detected using rabbit peroxidase ABC Kit (no. PK-6101; Vector Lab) according to the manufacturer’s instructions. Substrate was developed using NovaRed (no. SK-4800; Vector Lab) or DAB (no. SK4100, Vector Lab). Sections were counterstained with haematoxylin. For IF, sections were treated as before and after the secondary antibody incubation Fluorescein Avidin DCS (1:500 in PBS, no. A-2011, Vector Lab) was applied for 20 min. For IF on MAFs, Alexa Fluor secondary antibody (1:2,000; Molecular Probes) was applied for 30 min at room temperature. Sections or cells were stained with DAPI for 5–10 min and mounted in vectashield mounting media.

### Antibodies for IHC/IF

Anti-γ-H2A.X IgG (S139) (no. 9718, rabbit monoclonal, Cell Signaling, 1:250), anti-Ki-67 (no. ab15580, rabbit polyclonal, Abcam, 1:50), HNE (HNEJ-2) (no. MHN-100P, mouse monoclonal, Japan Institute for the Control of Ageing, Japan, 1:50), PCNA (no. ab29, mouse monoclonal, Abcam, 1:1,000), α-sma FITC (no. F3777, Sigma, 1:2,000), fluorescein isothiocyanate FITC (no. P510050-8, Dako, 1:2,000), polyclonal swine anti-rabbit biotinylated (no. E0353, Dako, 1:200), rat anti human CD3 (no. MCA1477, Serotec), goat anti rat biotinylated secondary antibody (no. STAR80B, Serotec, 1:200), 53BP1 (no. NB100-305, Novus Biologicals, 1:200).

### Antibodies for western blotting

Phospho-ATM (Ser 1981, no. AF1655 R&D, 1:250), ATM (no. 2873 Cell Signalling, 1:500), COX-2 (no. 12282 Cell Signalling, 1:250), phospho-p53 (Ser15, no. 9284 Cell Signalling, 1:1,000), p53 (no. 9282 Cell Signalling, 1:1,000) and GAPDH (no. 5174 Cell Signalling, 1:10,000).

Apoptotic cells were detected using ApopTag kit (no. S7100, Millipore). For Sen-β-gal staining^43^, cells grown on coverslips were fixed in 2% paraformaldehyde made up in PBS for 10 min at room temperature. The cells were washed 2 × 3 min in PBS and then stained at 37 °C for 24 h in Sen-β-Gal staining solution containing 2 mM magnesium chloride, 150 mM sodium chloride, 40 mM citric acid, 12 mM sodium phosphate dibasic, 5 mM potassium ferrocyanide, 5 mM potassium ferricyanide and 1 mg ml^−1^ 5-bromo-4-chloro-3-inolyl-β-D-galactoside (X-Gal) at pH 5.5. The cells were washed 2 × 3 min with PBS, counterstained with DAPI and mounted with vectashield. Epidermal thickness was measured on H&H sections from basement membrane to the start of the stratum corneum, ensuring measurements were taken in interfollicular spaces.

### Telomere ImmunoFISH

After γ-H2A.X IF, slides were washed three times in PBS, crosslinked with 4% paraformaldehyde for 20 min and dehydrated in graded ethanol. Sections were denatured for 10 min at 80 °C in hybridization buffer (70% formamide (Sigma), 25 mM MgCl_2_, 0.1 M Tris (pH 7.2), 5% blocking reagent (Roche)) containing 2.5 μg ml^−1^ Cy-3-labelled telomere-specific (CCCTAA) peptide nucleic acid probe (Panagene), followed by hybridization for 2 h at room temperature in the dark. Slides were washed twice with 70% formamide in 2 × SSC for 15 min, followed by washes in 2 × SSC and PBS for 10 min. Sections were incubated with DAPI, mounted and imaged. In depth Z stacking was used (a minimum of 40 optical slices with × 63 objective) followed by Huygens (SVI) deconvolution. Relative telomere length was measured by telomere intensity per nucleus in one *z* plane.

### *In vitro* crypt culture

Crypt isolation was carried out as previously described^66^. Crypts were then mixed with 50 μl of Matrigel (BD Biosciences) and plated in 24-well plates. The plate was then incubated at 37 °C for 15 min to allow the Matrigel to solidify. Crypt culture medium (500 μl; Advanced DMEM/F12, B27 and N2 supplement (Invitrogen), 1.25 mM N-acetylcysteine (Sigma), 50 ng μl^−1^ murine epidermal growth factor, 100 ng μl^−1^ murine Noggin (Peprotech), 500 ng μl^−1^ mouse recombinant R-Spondin 1 (R&D Systems) was added to each well. The number of crypts seeded per well was then quantified. The plate was then transferred to a BD Biosciences Biostation where 10 crypts were randomly chosen to be monitored every 6 h for 10 days to obtain growth curves. Crypt culture medium was changed every 2 days and total organoid growth frequency was quantified after 10 days.

### Statistical analysis

Single comparisons were performed using two-tailed Student’s *t*-test and multiple comparisons by one-way ANOVA followed by *post hoc* all pairwise multiple comparisons (Holm–Sidak). For survival analysis, Kaplan–Meier log-rank analysis (right-censored) was performed.

## Author contributions

D.J. performed the majority of experiments and contributed to study design; C.W., J.F.P., F.O., C.C.-M., L.G., G.S., C.F., C.L., R.A., G.H., N.F., G.N. and J.M. performed and evaluated individual experiments; S.L.F.P. and B.v.d.S. provided materials; D.A.M. and T.v.Z. designed and supervised the study; T.v.Z. wrote the paper with contributions from D.J., J.F.P., F.O. and D.A.M.

## Additional information

**How to cite this article:** Jurk, D. *et al*. Chronic inflammation induces telomere dysfunction and accelerates ageing in mice. *Nat. Commun.* 5:4172 doi: 10.1038/ncomms5172 (2014).

## Supplementary Material

Supplementary Information Supplementary Figures 1-7

## Figures and Tables

**Table 1:** Pro-inflammatory phenotypes in wt and *nfkb1* ^*−/−*^ mice.

**Phenotype**	***nfkb1***^*−/−*^ **(12 weeks)**	***nfkb1***^*−/−*^ **(36 weeks)**	**wt (12 weeks)**	**wt (36 weeks)**	**wt (104 weeks)**	**wt (132 weeks)**
Enlarged spleen*	3/5	6/6	0/5	0/5	1/7	4/5
High systemic IL-6†	0/5	5/5	0/4	2/5	n.d.	5/5
Liver: immune cell infiltration‡	1/5	5/5	0/5	0/5	n.d.	2/3

Data are no. of animals showing the phenotype/no. of animals observed.

^*^Spleen mass >0.35% of body mass

^†^>6 pg ml^−1^

^‡^>6 CD3^+^ cells per field

**Table 2:** Ageing-associated phenotypes in wt and *nfkb1^−/−^* mice.

**Phenotype**	***nfkb1***^*−/−*^ **(36 weeks)**	**wt (36 weeks)**	**wt (104 weeks)**	**wt (132 weeks)**
Low coat quality score*	8/14	0/15	7/12	4/6
Low body condition score†	13/14	3/15	9/12	5/6
Low welfare status score‡	7/14	0/15	9/12	4/6
Ataxia	1/14	1/15	3/12	3/6
kyphosis	0/9	0/15	0/12	0/6
sarcopenia§	0/6	0/6	2/6	4/4
Terminal weight loss||	3/8	0/24	6/19	8/9
Low total body fat¶	6/6	0/5	0/5	4/5
Low subcutaneous fat#	5/6	0/5	3/7	5/5
Cardiac hypertrophy**	3/6	0/5	0/6	4/5
Liver: lipofuscin	2/3	1/8	1/4	6/6
Liver: karyomegaly††	4/5	1/5	3/3	3/3
Brain: lipofuscin	2/5	0/7	n.d.	4/4
Macroscopic tumours	0/6	0/7	3/7	7/11
death	6/23	0/52	15/52	34/52

Data are no. of animals showing the phenotype/no. of animals observed.

^*^Alopecia, hair greying, piloerection, fur roughness scored at a scale from 1 to 10, scores below 7 are counted as low.

^†^Body condition^67^ scored at a scale from 1 to 10, scores below 7 are counted as low.

^‡^Physical, physiological and psychological status scored according to Hawkins *et al*.^68^ at a scale from 1 to 10, scores below 7 are counted as low.

^§^Mean hind leg muscle fibre diameter <36 μm

^||^Weight >3 g below max for >1 month

^¶^Total body fat=Sum (inguinal+epidymal+perirenal+mesenteric) <5% of BW

^#^Subcutaneous fat <0.7 g

^**^Heart mass >0.55% of BW

^††^>10% of hepatocyte nuclei
