# Supplementary material for: Chronic inflammation induces telomere dysfunction and accelerates ageing in mice
Source: Nat Commun. 2014 Jun 24;2:4172. doi: 10.1038/ncomms5172 (PMC4090717; doi:10.1038/ncomms5172)
Supplement: Supplementary Information — Supplementary Figures 1-7 [file ncomms5172-s1.pdf]

Supplemental Figures

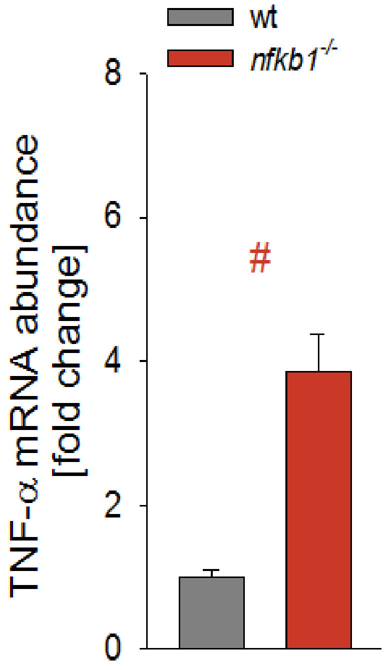

Supplementary Fig. 1: Relative TNFα mRNA abundance in livers from wt and nfkb1$^{-/-}$ at 12 weeks of age. Data are mean ± SEM, n=5, p = 0.00077, t test.

a

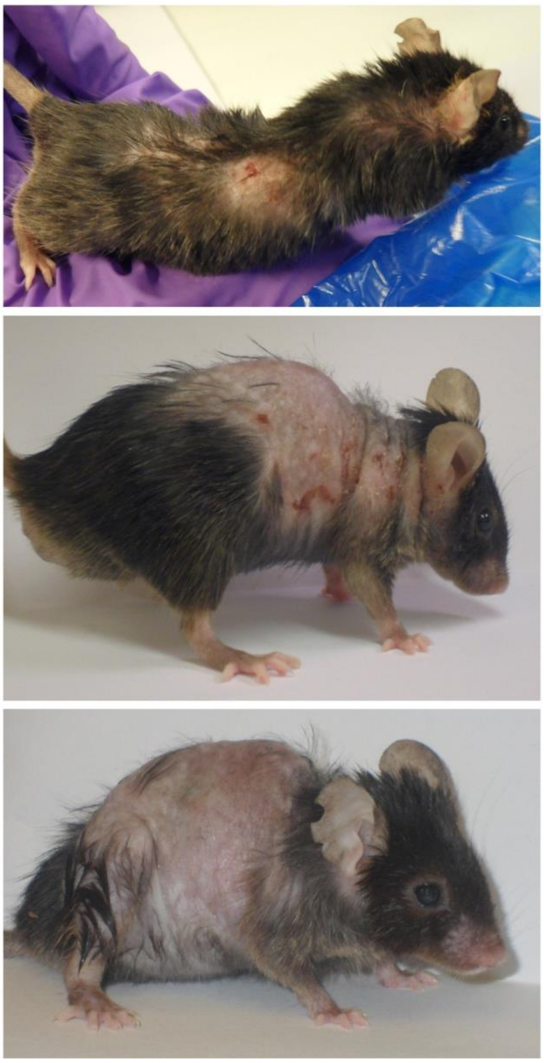

b

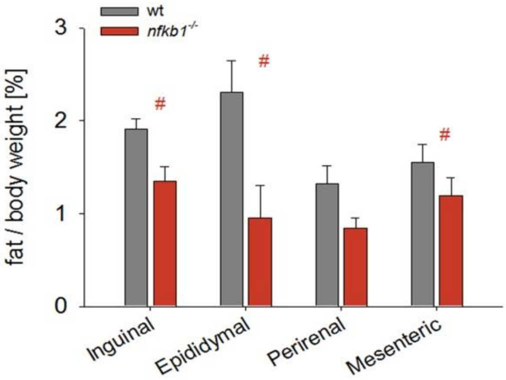

Supplementary Fig. 2: Additional premature aging phenotypes in nfkb1$^{-/-}$ mice. a) Range of phenotypic variation in *nfkb1*$^{-/-}$ mice older than 44 weeks of age. All *nfkb1*$^{-/-}$ mice in this age group show premature hair greying and scruffy fur. A fraction of mice develop hair loss and skin inflammation (top) and in more extreme cases, hair loss is accompanied by kyphosis (middle and bottom). **b)** Body mass normalized fat depot size in wt and *nfkb1*$^{-/-}$ mice at 36 weeks of age (n=6 for *nfkb1*$^{-/-}$ and n=5 for wt, t test).

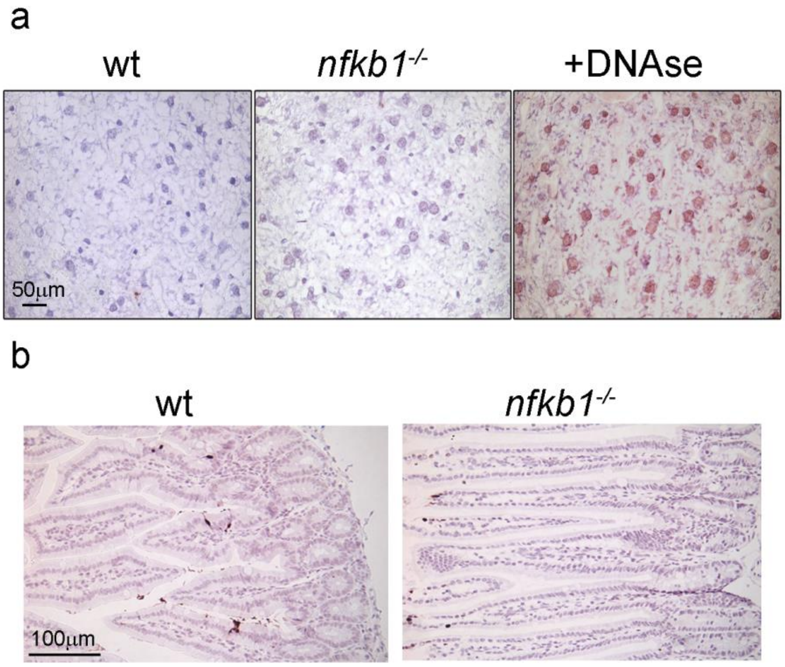

Supplementary Fig. 3: Decreased regenerative capacity in *nfkb1*$^{-/-}$ mice is not associated with increased apoptosis. a) Representative Apoptag images of wt and *nfkb1*$^{-/-}$ livers from 36 weeks old mice. DNase treatment is included as positive control (right). **b)** Representative Apoptag images of wt and *nfkb1*$^{-/-}$ intestines from 36 weeks old mice. Very few apoptotic enterocytes are seen except at the villus tips.

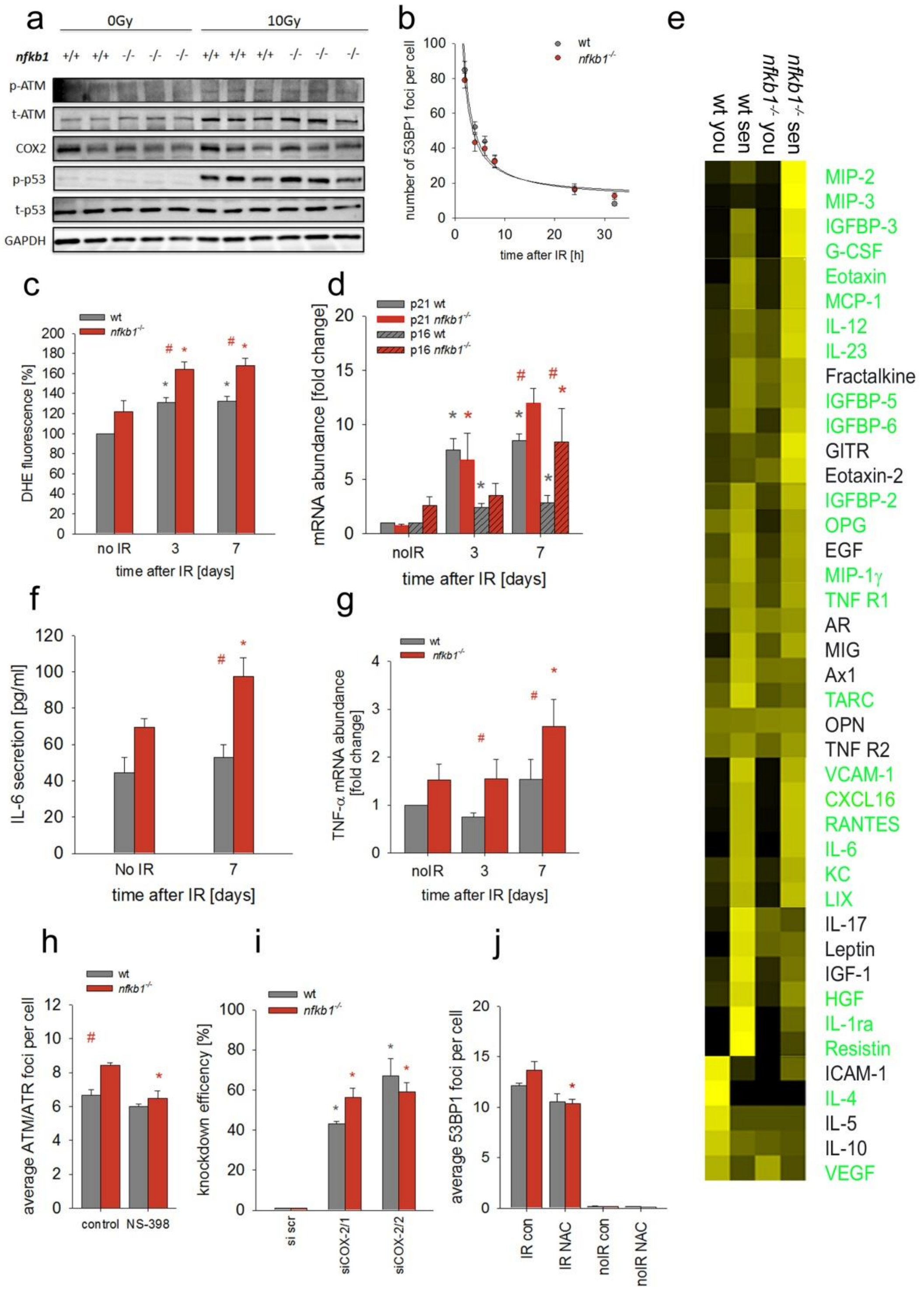

Supplementary Fig. 4: The senescent phenotype is aggravated in *nfkb1*$^{-/-}$ MAFs. Data are M ± SEM from 3 independent strains per genotype. Significant differences (ANOVA with post-hoc Holm-Sidak test, $p<0.05$) to respective controls of the same genotype are indicated by *, and between wt and *nfkb1*$^{-/-}$ strains at the same time point by #. **a)** Western blots of the indicated proteins in MAFs at 4h after 10 Gy IR. Full blots are shown as Supplementary Fig. 7. **b)** Frequencies of nuclear 53BP1 foci in MAFs at the indicated times after 10 Gy IR. **c)** Cellular ROS levels in MAFs after senescence induction measured by DHE fluorescence in flow cytometry. **d)** Transcription of cell cycle

inhibitors CDKN1A and CDKN1B is enhanced in *nfkb1*$^{-/-}$ MAFs in stress-induced senescence. CDKN1A (p21) and CDKN2A (p16) mRNA abundance measured by qPCR. **e)** The SASP is stronger in *nfkb1*$^{-/-}$ MAFs. Quantitative secreted chemokine array (means of 4 independent strains per condition) from proliferation-competent (you) and senescent (sen) wt and *nfkb1*$^{-/-}$ MAFs. Significant differences (ANOVA) are indicated in green. **f)** IL-6 secretion from wt and *nfkb1*$^{-/-}$ MAFs measured by ELISA in the cell culture supernatant. **g)** TNF-α mRNA abundance measured by qPCR is enhanced in *nfkb1*$^{-/-}$ MAFs in stress-induced senescence at the indicated times after senescence induction**. h)** ATM/ATR foci frequencies in MAFs treated or not with the COX-2 inhibitor NS-398 at 10 d after IR. **i)** Knock-down efficiency of 2 separate siRNAs against COX-2. Knockdown was measured in irradiated MAFs by quantitative RT-PCR using β–Actin as reference. **j)** The antioxidant NAC reduces nuclear 53BP1 foci frequencies in *nfkb1*$^{-/-}$ MAFs. MAFs were irradiated (or not) with 10Gy and treated (or not) with 5 mM NAC for 3 days.

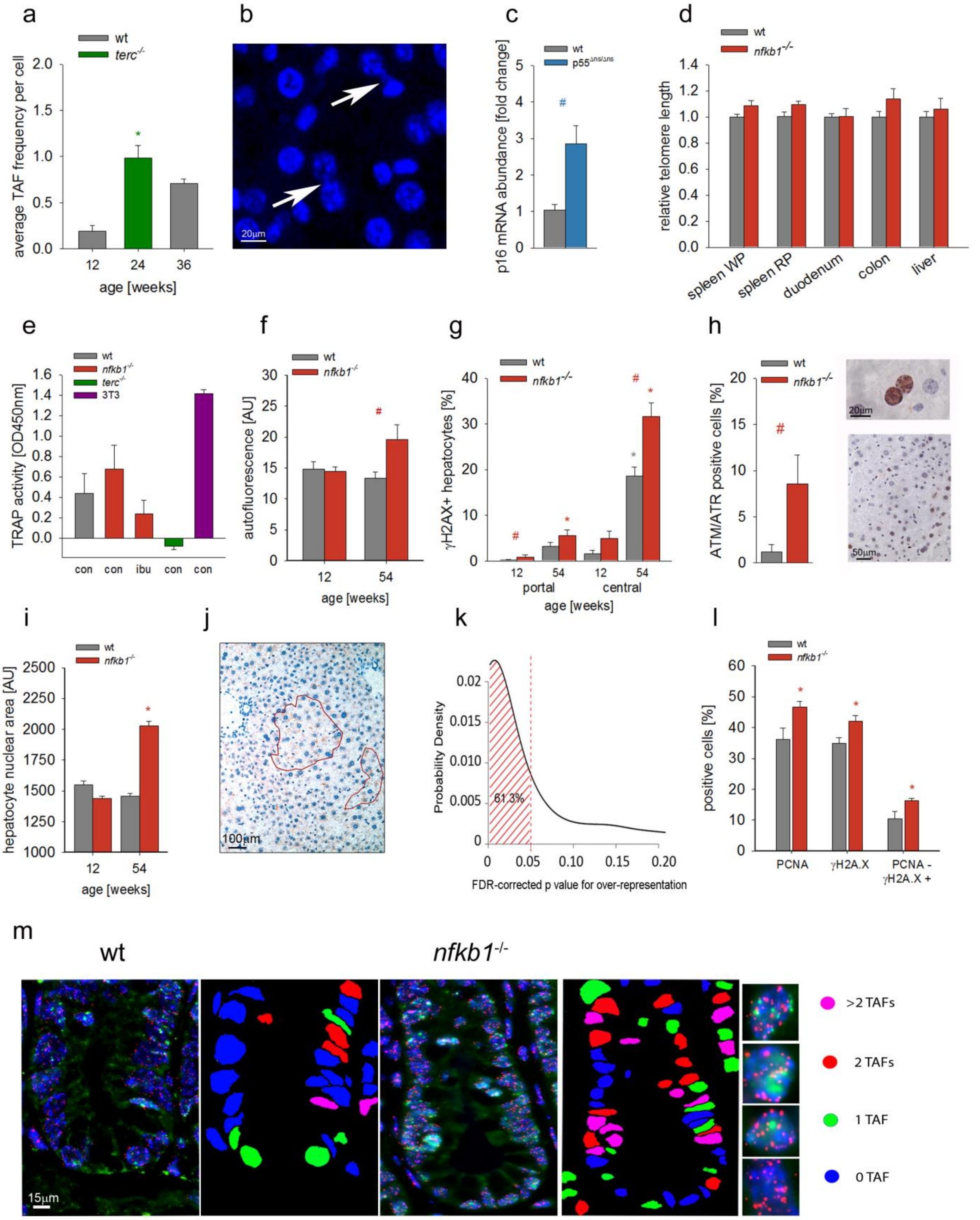

Supplementary Fig. 5: Additional markers of senescence in mice tissues. Data are M ± SEM from 4-6 animals per group, if not indicated otherwise. Significant differences (ANOVA with post-hoc Holm-Sidak test, $p<0.05$) to respective controls of the same genotype are indicated by *, and between wt and *nfkb1*$^{-/-}$ strains at the same time point by #. **a)** TAF frequencies per nucleus in livers from late generation *terc*$^{-/-}$ and wt mice at the indicated ages. TAF frequencies in *terc*$^{-/-}$ livers at 24 weeks of age are already significantly higher (ANOVA with post-hoc Holm-Sidak test, $p<0.05$) than those in wt livers at 36 weeks. **b)** Representative DAPI image of *nfkb1*$^{-/-}$ liver at 72h after partial hepatectomy.

Arrows indicate chromatin bridges between nuclei. **c)** Relative p16 mRNA abundance in livers from p55$^{\Delta ns/\Delta ns}$ and control mice. **d)** Relative telomere length in tissues from *nfkb1*$^{-/-}$ and control mice. Differences between wt and *nfkb1*$^{-/-}$ are not significant. **e)** Telomerase activity (TRAP) activity in liver lysates (1 μg protein). *terc-/-* lysates are included as negative control, and 3T3 cell lysates (100 ng) as positive control. *nfkb1-/-* mice were 32 weeks old, treated or not for 8 weeks with ibuprofen. Unadjusted p values (ANOVA) are 0.336 for wt vs *nfkb1-/-* and 0.087 for *nfkb1*$^{-/-}$ vs *nfkb1*$^{-/-}$ ibu. **f)** Autofluorescence intensity in wt and *nfkb1*$^{-/-}$ livers. **g)** Frequencies of γ-H2A.X positive, PCNA negative hepatocytes in periportal and centrilobular areas of the liver. **h)** Frequencies of ATM/ATR-positive hepatocytes in wt and *nfkb1*$^{-/-}$ livers at 36 weeks of age ($p<0.05$, t test). **i)** Hepatocyte nuclear area increases in aged *nfkb1*$^{-/-}$ livers. Data are M ± SEM from at least 600 nuclei per group. Comparisons between genotypes at the same age were done by t test. **j)** Representative tiled 4-HNE immunohistochemistry image of *nfkb1*$^{-/-}$ liver. Clusters of adjacent 4-HNE+ hepatocytes are marked by red lines. **k)** Cluster probability vs. p value for over-representation corrected for multiple comparisons by false discovery rate (FDR) control. The fraction of positive cells having significantly more positive neighbours than expected by chance given the background proportion of positive cells is indicated. **l)** Frequencies of PCNA+, γH2AX+ and PCNA-γH2AX+ enterocytes in wt and *nfkb1*$^{-/-}$ intestines at 36 weeks of age. Comparisons between genotypes were done by t test. **m)** Representative immunoFISH images (deconvolved maximum intensity projections) from wt and *nfkb1*$^{-/-}$ intestinal crypts at 36 weeks of age. TAF were counted using whole image stacks. In the masks, nuclei are colour-coded according to their TAF frequencies (pink > 2 TAF, red = 2 TAF, green = 1 TAF, blue = 0 TAF). Examples of individual nuclei with increasing TAF frequency are shown on the right.

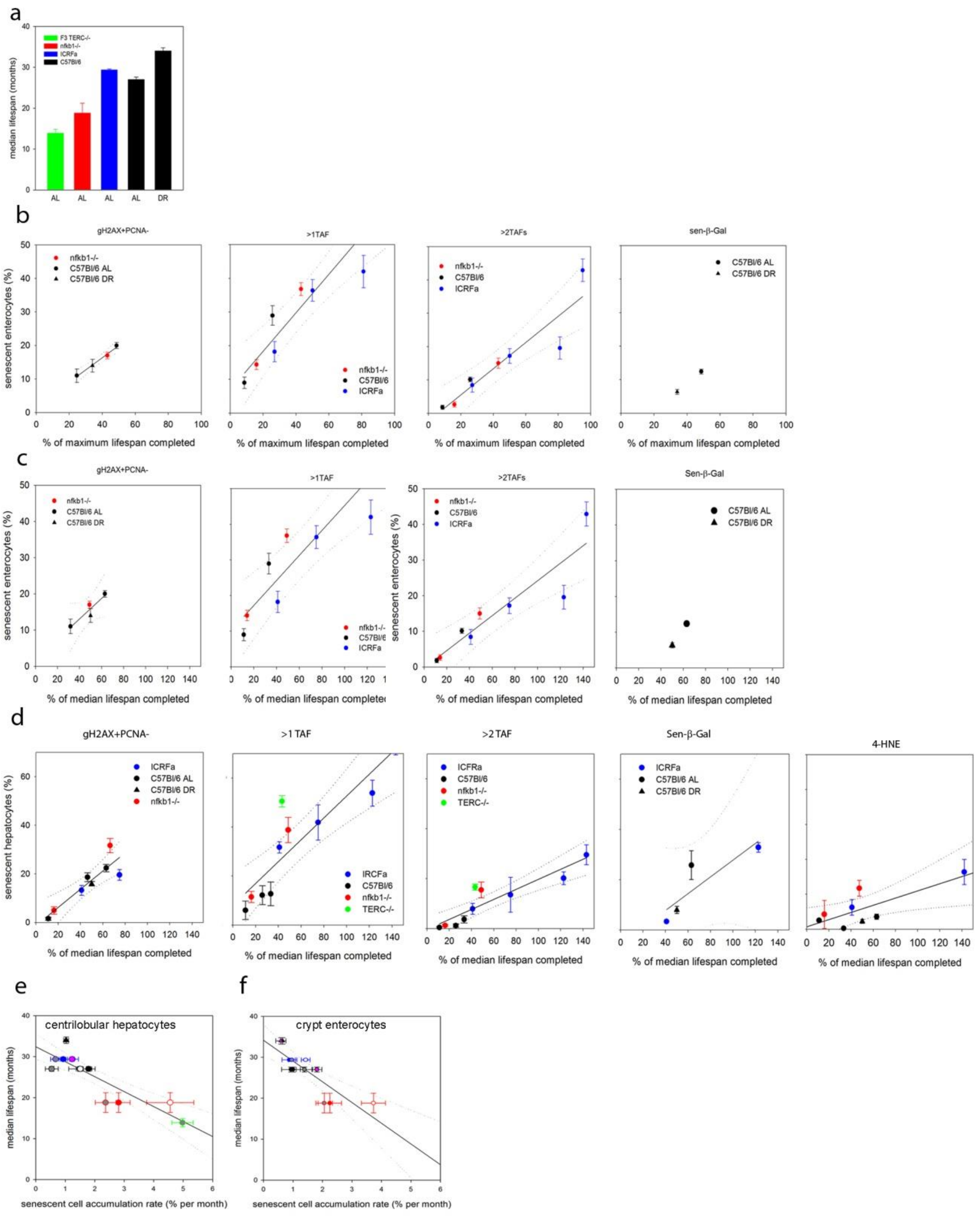

Supplementary Fig. 6: Linear correlation between senescent cell frequencies and relative age in different mice strains. a) Median lifespan in the indicated strains of mice under ad libitum feeding (AL) and dietary restriction (DR). Kaplan-Meyer survival analysis, censored data. **b)** Frequencies of senescent crypt enterocytes measured as γH2AX+PCNA- cells (left), cells with >1 TAF (middle left), cells with >2TAF (middle right) or Sen-β-Gal+ cells (right) versus relative age (as % of maximum lifespan completed) in the strains as indicated by symbol colours. Triangle indicates a strain under dietary restriction. Data are M±SEM from at least 3 mice per age group and condition. **c)** Frequencies of senescent crypt enterocytes vs % of median lifespan completed in the indicated strains. Data are as in (b). **d)** Frequencies of senescent hepatocytes vs % of median lifespan

completed in the strains as indicated. Data are as in (b) but with 4-HNE added as additional marker (far right). **e)** and **f)** Accumulation rate of senescent cells in liver **(e)** and intestinal crypts **(f)** predicts median lifespan. Shown are rates of accumulation of senescent hepatocytes **(e)** or crypt enterocytes **(f)**, calculated by linear regression against age as independent variable and median lifespan per strain/condition as dependent variable. Symbol outline colours indicate strain/condition (as above). Symbol fills indicate the senescence marker used: outline colour: γH2AX+PCNA-, pink: Sen-β-Gal, white: >1TAF, grey: >2TAFs.

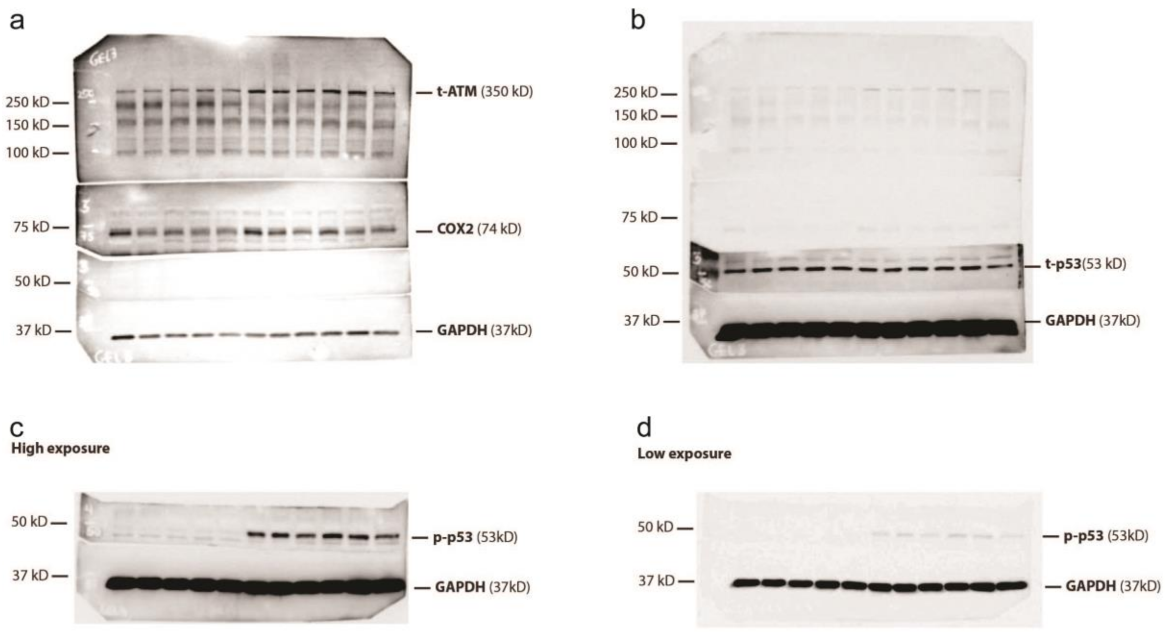

Supplementary Fig. 7. Full blots including size marker positions for the most important proteins shown in Supplementary Fig. 4a. a) Membrane blotted with antibodies against t-ATM, COX2, t-p53 and GAPDH. Concentration of anti-p53 antibody was too low. **b)** Same membrane re-blotted with higher concentration of anti-p53 antibody and longer exposure. **c)** and **d)** Different exposures of membrane blotted with anti-p-p53 and GAPDH antibodies.
